# Fluorescence-readout as a powerful macromolecular characterisation tool

**DOI:** 10.1039/d3sc04052f

**Published:** 2023-10-20

**Authors:** Xingyu Wu, Christopher Barner-Kowollik

**Affiliations:** a School of Chemistry and Physics, Centre for Materials Science, Queensland University of Technology (QUT) 2 George Street Brisbane QLD 4000 Australia christopher.barnerkowollik@qut.edu.au; b Institute of Nanotechnology (INT), Karlsruhe Institute of Technology (KIT) Hermann-von-Helmholtz-Platz 1 76344 Eggenstein-Leopoldshafen Germany

## Abstract

The last few decades have witnessed significant progress in synthetic macromolecular chemistry, which can provide access to diverse macromolecules with varying structural complexities, topology and functionalities, bringing us closer to the aim of controlling soft matter material properties with molecular precision. To reach this goal, the development of advanced analytical techniques, allowing for micro-, molecular level and real-time investigation, is essential. Due to their appealing features, including high sensitivity, large contrast, fast and real-time response, as well as non-invasive characteristics, fluorescence-based techniques have emerged as a powerful tool for macromolecular characterisation to provide detailed information and give new and deep insights beyond those offered by commonly applied analytical methods. Herein, we critically examine how fluorescence phenomena, principles and techniques can be effectively exploited to characterise macromolecules and soft matter materials and to further unravel their constitution, by highlighting representative examples of recent advances across major areas of polymer and materials science, ranging from polymer molecular weight and conversion, architecture, conformation to polymer self-assembly to surfaces, gels and 3D printing. Finally, we discuss the opportunities for fluorescence-readout to further advance the development of macromolecules, leading to the design of polymers and soft matter materials with pre-determined and adaptable properties.

## Abstract 2

We discuss the current state-of-the-art in exploiting fluorescence-based techniques for analysing polymer systems and soft matter materials, as well as provide an outlook on future opportunities and challenges.

## Graphical abstract

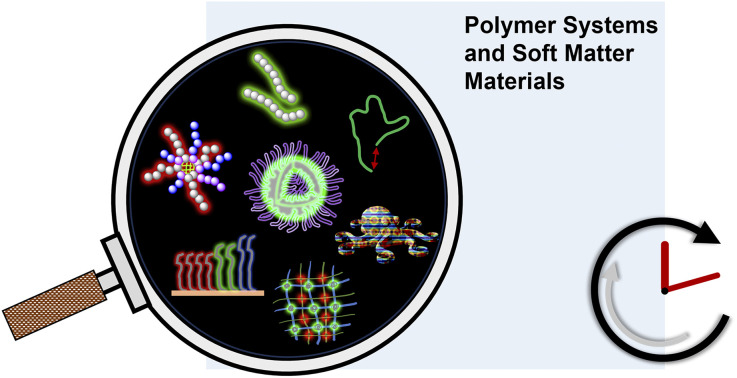

## Introduction

Polymers play an indispensable and ubiquitous role in human society, with applications ranging from clothing to medication to aviation. Polymers are formed through polymerisation processes, which transform monomers into polymeric chains. During the last few decades, polymerisation processes have matured enormously – especially benefitting from a fusion with advanced organic chemistry methodologies – and it is perhaps no exaggeration to note that almost any polymer topology and functionality is accessible within certain limits, including in the by now established field of sequence-defined polymers.^1–9^ Historically, the discovery of living anionic polymerisation by Michael Szwarc in 1956 opened a key avenue for the synthesis of well-defined polymers,^10^ albeit not with the precision of contemporary sequence-defined polymers. Subsequently, the advent of reversible deactivation radical polymerisation (RDRP),^11–19^ ring-opening polymerisation,^20–22^ ring-opening metathesis polymerisation (ROMP)^23–25^ has revolutionised polymer science, enabling the above noted complex macromolecular architectures, including self-assembly through non-covalent linking (including supramolecular interactions), enabling applications in nanocomposites to nanomedicine.^5,26–37^ In order to perform polymerisation under more diverse conditions to address a broader scope of applications, light,^38–42^ electricity,^43–45^ mechanical force^46,47^ and chemical triggers^48,49^ have been successfully utilised as external regulators in polymerisation reactions. Among them, light is arguably the most attractive regulator due to its unique capability to spatiotemporally control chemical reactions. In particular, photo-controlled RDRP and light triggered ligation techniques have enabled advanced surface fabrication and new 3D printing methodologies, providing materials with advanced properties, such as self-healing, stimuli-responsiveness and reversibility.^50–58^ Very recently, flow technology has received significant attention in polymer synthesis to improve reproducibility, facilitate high-throughput synthesis, and even achieve autonomous self-optimisation and self-control.^59–62^

These significant developments have brought us closer to achieving precise control over soft matter material properties on the molecular level.^37,63–65^ To reach this aim, it is critical to develop advanced analytical techniques able to investigate polymers and polymerisation processes at molecular and microscopic levels, in addition to progressing advanced synthetic methods. A plethora of analytical techniques are routinely applied in polymer science, including spectroscopic methods (nuclear magnetic resonance (NMR) spectroscopy, mass spectrometry (MS), ultraviolet-visible (UV-Vis) spectroscopy , Fourier-transform infrared spectroscopy (FTIR), Raman spectroscopy, X-ray photoelectron spectroscopy (XPS)), thermal and mechanical methods (differential scanning calorimetry (DSC), dynamic mechanical analysis (DMA)), chromatography (size-exclusion chromatography (SEC), high-performance liquid chromatography (HPLC)), gravimetry, microscopy (optical microscopy, atomic force microscopy (AFM), scanning electron microscopy (SEM), transmission electron microscopy (TEM)) as well as coupled techniques – all of which are able to provide valuable information, such as molecular weight, molecular weight distributions, chemical compositions and polymer structures. However, when considering the time scale of polymerisation processes and dynamics in polymer systems, the complexity of polymer architectures and organisation as well as heterogeneities of polymer systems, these techniques exhibit limitations and disadvantages arising from the necessity of sample preparation, invasive and destructive tests, low sensitivity and macroscopic measurements. Thus, it is a considerable challenge for polymer scientists to investigate polymer systems and polymerisation processes at molecular and microscopic levels, especially during the macromolecular growth process.

Among the available methodologies, fluorescence-based techniques present appealing features, *i.e.*, high sensitivity (*e.g.*, sub-micrometre spatial resolution and below millisecond time resolution), high selectivity, large contrast, fast and real-time response, and non-invasive characteristics. The combination of fluorescence techniques, such as steady-state, time-resolved fluorescence spectroscopy, and fluorescence microscopy,^66–71^ with various fluorescence principles, including Förster resonance energy transfer (FRET)^72,73^ and aggregation-induced emission (AIE)^74,75^ can provide outstanding opportunities for polymer chemists and physicists to investigate polymer systems and polymerisation processes.^76–85^ For example, fluorescence spectroscopy can provide information on solvent relaxation in polymer systems, dynamics in macromolecular self-assemblies and physical aging of polymers in timescales varying from picoseconds up to years;^86–89^ fluorescence-imaging microscopy can give access to 3D-structural and morphological information of macromolecular ensembles down to a single molecule.^89–93^

In fact, fluorescence-based techniques have been applied to analyse polymers for several decades. In the current review, we will not address the related research before 2010 and refer the reader to the relevant literature.^89,94–96^ However, significant advances have been made thereafter regarding advanced polymer synthetic techniques and their applications, such as photo-controlled RDRP,^42^ polymerisation-induced self-assembly (PISA),^97^ supramolecular polymerisation,^98^ sequence-controlled polymerisation,^1^ surface-initiated controlled radical polymerisation (SI-CRP)^99^ and 3D and 4D printing.^100–103^ In addition, recent years have also seen exciting advances in exploiting fluorescence principles and technologies in synthetic polymer systems, including fluorogenic,^104^ AIE,^105^ FRET,^82^ fluorescence lifetime imaging microscopy (FLIM),^85^ stimulated emission depletion microscopy (STED)^106,107^ and single-molecule localisation microscopy (SMLM).^83,92,93^ Thus, our aim is to provide a contemporary review that critically assesses the recent advances in the field and the opportunities and challenges that have emerged.

**Fig. 1:**
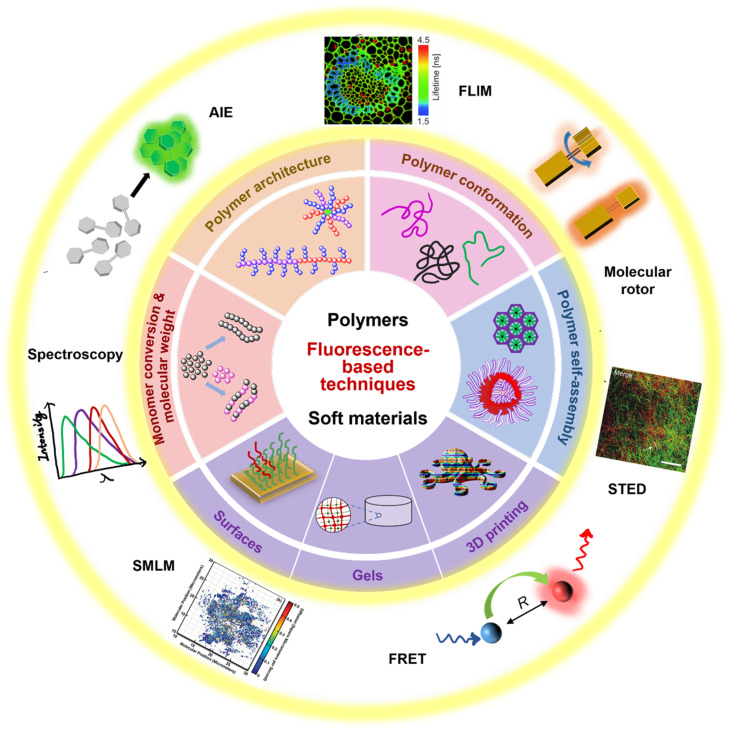
Summary of fluorescence principles (*e.g.*, molecular rotor, aggregation-induced emission (AIE), Förster resonance energy transfer (FRET)) and techniques (*e.g.*, fluorescence spectroscopy, fluorescence lifetime imaging microscopy (FLIM), stimulated emission depletion (STED) and single molecule localisation microscopy (SMLM)) employed to fundamental areas of polymer science. STED image reproduced from ref. 107 with permission from Springer Nature, copyright 2016. FLIM image copyright by PicoQuant GmbH. SMLM image copyright by ZEISS.

In the current review, we thus focus on recent scientific achievements covering the analysis of wide aspects of synthetic polymer systems based on fluorescence techniques (refer to Fig. 1). We provide an overview of the field *via* the discussion of representative examples from 2010 onwards. The review is organised as follows: we commence with two essential parameters for polymerisation processes – monomer conversion and molecular weight, describing different fluorescence phenomena and their adaptation to various types of polymerisations for the determination of these two parameters. We subsequently discuss fluorescence-based techniques for the investigation of polymer architecture, conformation and self-assembly, highlighting the use of a variety of fluorescence phenomena and advanced technical methods to access different parameters in these areas, for example the lengths of arms in specific polymer architectures, end–end distances in polymer conformations as well as monomer exchange dynamics during self-assembly. Extending from polymers to soft matter materials, next, we discuss how fluorescence-based techniques can benefit the study of polymer surfaces, gels and 3D-printed structures. Finally, we share our vision for the future of exploiting fluorescence-based techniques in polymer and materials science, which we suggest will give further deep and new insights.

## Monomer conversion and molecular weight

In polymer chemistry, monomer conversion is defined as the consumption of the initial monomer(s) during macromolecular growth, and molecular weight often refers to an average molecular weight due to dispersity. Both of these are strongly linked to the progress of polymerisation and have a striking impact on the polymer properties, from the rheological and mechanical properties to morphological characteristics.^108,109^ Therefore, following the reaction kinetics *via* monomer conversion and determining the molecular weight of a polymer is often the initial key step in its characterisation.

Monomer conversion and molecular weight can be determined by several analytical methods, including gel-permeation chromatography/size-exclusion chromatography (GPC/SEC), NMR spectroscopy, MS, FTIR and Raman spectroscopy. However, these methods are typically challenged in providing real-time information for ongoing reactions, and they often require fully soluble polymers, preventing characterisation of cross-linked and high-molecular weight polymers as well as conjugated polymers. Fluorescence-based techniques provide the opportunity to overcome these difficulties. In order to successfully visualise and monitor polymerisation processes, the addition of a fluorophore (profluorophore) to a polymer system or, in many cases, the covalent attachment of a fluorophore (profluorophore) to a polymer chain is often required due to the generally very weak intrinsic fluorescence of polymers.

In the following, a variety of fluorophores will be introduced with regard to different polymerisation systems ranging from conventional polymerisation, conventional photopolymerisation, to controlled polymerisation, then to supramolecular polymerisation.

**Fig. 2:**
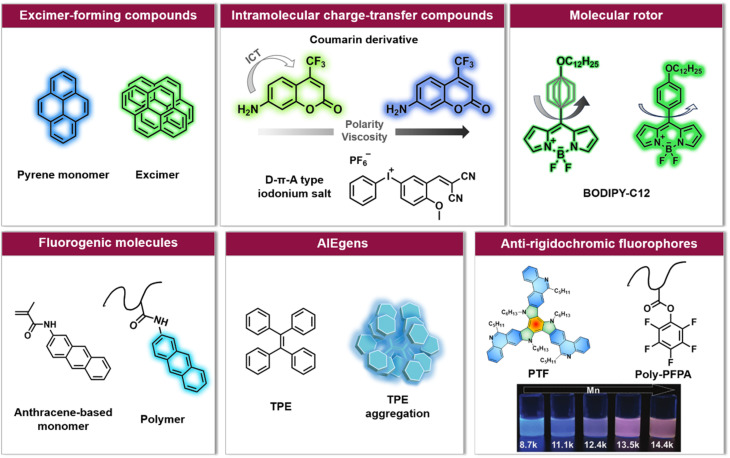
Overview of six categories of fluorescence compounds allowing to monitor the polymerisation process and map monomer conversion and molecular weight. Anti-rigidochromic fluorophores: images reproduced from ref. 141 with permission from Wiley, copyright 2022.

While different types of polymerisations exhibit different mechanisms and kinetics, the polymerising system always undergoes a transformation from a lower viscosity liquid to a more viscous, even rigid material as monomer conversion and molecular weight increases. Driven by this realization, various fluorophores (refer to Fig. 2) have been designed and applied to monitor conventional and photo-induced polymerisations.^110–113^

For example, pyrene can undergo intermolecular interactions to form excimers (dimers that exhibit different fluorescence emission from the single molecule, Fig. 2) depending on the lateral diffusion of pyrene in the medium.^114^ An increase in local viscosity limits the diffusion of the ground state pyrene molecules towards the excited state ones, resulting in a decrease in the ratio between the maximum fluorescence intensity of the excimer and the maximum fluorescence intensity of pyrene (*I*_excimer_/*I*_pyrene_). This has been exploited to follow the conversion of methyl methacrylate (MMA) *in situ* during miniemulsion polymerisation.^110^ However, a high concentration of pyrene is required in order to achieve sufficient proximity for two molecules to form excimers, and due to the rapid decrease of *I*_excimer_/*I*_pyrene_, it is often challenging to monitor long-term polymerisation processes.

Unlike the excimer-forming compounds, different types of environment-sensitive fluorophores including intramolecular charge transfer compounds (Fig. 2) have been developed by the group of Ortyl and others, such as coumarin-based molecules,^111,115^ 2-amino-4,6-diphenyl-pyridine-3-carbonitrile derivatives,^116^ rare earth complex compounds,^117^ meta-terphenyls,^118^ dansyl hyperbranched fluorophores,^119^ benzylidene scaffold-based and 4,4-difluoro-4-bora-3a,4a-diaza-*s*-indacene (BODIPY)-based iodonium salts,^112,120^ to monitor free-radical photopolymerisation, ring-opening and chain growth cationic photopolymerisation, based on a changing fluorescence intensity or fluorescence intensity ratio as well as shifting emission wavelengths. We note common principles: (i) when the excited state of the fluorophore is more polar than the corresponding ground state, the reduced polarity of the system leads to an increased energy gap between the ground and the excited state of the fluorophore. As a result, the fluorescence emission wavelength shifts toward shorter wavelengths; (ii) the increased microviscosity of the system slows the solvation of the excited fluorescent molecules, thus preventing them from reaching their most relaxed conformation before emission of a photon, while the rigidity of the medium inhibits conformational changes of the excited molecules which is one of the de-excitation pathways. Therefore, a hypsochromic shift of the emission wavelength and an increase in fluorescence intensity can be observed.^111,121^

Photo-induced polymerisation is now routinely used in coatings, adhesives, photolithography and 3D printing.^102,122–124^ One of the major benefits of photopolymerisation is the ability to manipulate the light source to switch reactions between ‘ON’ and ‘OFF’ states and to adjust polymerisation rates. In general, the same wavelength is applied for the initiation of the reaction and the excitation of the fluorophore. A very elegant approach is to use the existing photoinitiator in the system as a fluorophore for monitoring, such as benzylidene scaffold-based and BODIPY-based iodonium salts developed by Ortyl's group or 2,6-bis(furan-2-ylmethylidene) cyclohexan-1-one developed by Li *et al.*^112,120,125^ It is worth noting that the design of fluorophores for cationic polymerisation is more challenging as they need to resist strong acidic environments.

An alternative type of fluorophore is the molecular rotor (Fig. 2), whose fluorescence depends on the viscosity of the surrounding medium. Upon excitation, the molecular rotor enables rapid non-radiative de-excitation *via* intramolecular rotation in low viscosity environments, in contrast, high local viscosity hinders the rotation and thus limits non-radiative pathways, resulting in higher fluorescence intensities and quantum yields as well as longer fluorescence lifetimes.^126,127^ Molecular rotors, such as BODIPY-C12 (chemical structure refer to Fig. 2), are thus well suited to investigate polymerisation processes.^110,113,128^

For example, Nolle *et al.* have successfully used BODIPY-C12 to monitor bulk radical polymerisation of MMA.^113^ Different from the abovementioned studies, the measurements in their study were based on fluorescence lifetime rather than fluorescence intensity. Fluorescence lifetime detection is independent of the dye concentration, while this factor strongly affects fluorescence intensity measurements.^129,130^ Importantly, the evolution of heterogeneities during the polymerisation process was elucidated in their study. Meanwhile, the fluorescence lifetimes were obtained from an advanced fluorescence technique – FLIM.^66,131^ In addition to the values of fluorescence lifetime, FLIM can provide spatially resolved images for additional information, which will be discussed later. Besides, the choice of BODIPY-C12 allows for the measurement of local viscosity. Unlike the previously mentioned fluorescent molecules and many other molecular rotors, whose fluorescence is strongly influenced by polarity and temperature, the fluorescence of BODIPY-C12 is only very weakly affected by these factors, at least at high viscosities.^129,132^

In addition to these three types of fluorophores, fluorogenic molecules, aggregation-induced emission luminogens (AIEgens) and others (Fig. 2) have also been applied to visualise the polymerisation process,^133–135^ mainly controlled polymerisation. Controlled polymerisation – especially RDRP – enables the synthesis of polymers with pre-determined average molar masses, narrow molecular weight distribution, diverse compositions and well-defined architectures. RDRP has driven the rapid advance of polymer science and benefited numerous fields, including biomedicine, energy and nanotechnology.^5,26^ Recently, the utility of RDRP has been further extended by various externally regulated polymerisations, such as photo-controlled RDRP.^42^ In all RDRPs, the establishment of a dynamic equilibrium between propagating radicals and various dormant species is central,^26^ and has been achieved through different methods, including atom transfer radical polymerisation (ATRP),^15,16^ nitroxide-mediated radical polymerisation (NMP),^13,14^ and reversible addition–fragmentation chain-transfer (RAFT) polymerisation.^17–19^

Allen *et al.* have synthesised fluorogenic monomers to *in situ* monitor ATRP in aqueous media.^133^ These monomers are meth-acrylamide derivatives of polycyclic aromatic hydrocarbon (PAH) probes such as pyrene, anthracene and acridine (Fig. 2). After their incorporation into polymer chains, these originally non-fluorescent PAH probes will become fluorescent, and the fluorescence intensity of the polymer system increases as a function of reaction time. A similar working principle has been applied by our group to photoinduced nitrile imine-mediated tetrazole-ene cycloaddition (NITEC) step-growth polymerisation, which represents one of the few examples of a photochemically driven step-growth polymerisation.^134^ The reactions between the non-fluorescent tetrazole moiety and the non-fluorescent dialkenes produce the fluorescent pyrazoline-containing polymer. Thus, the fluorescence emission directly correlates to the number of ligation points in the polymer, forming an ideal self-reporting system.

**Fig. 3:**
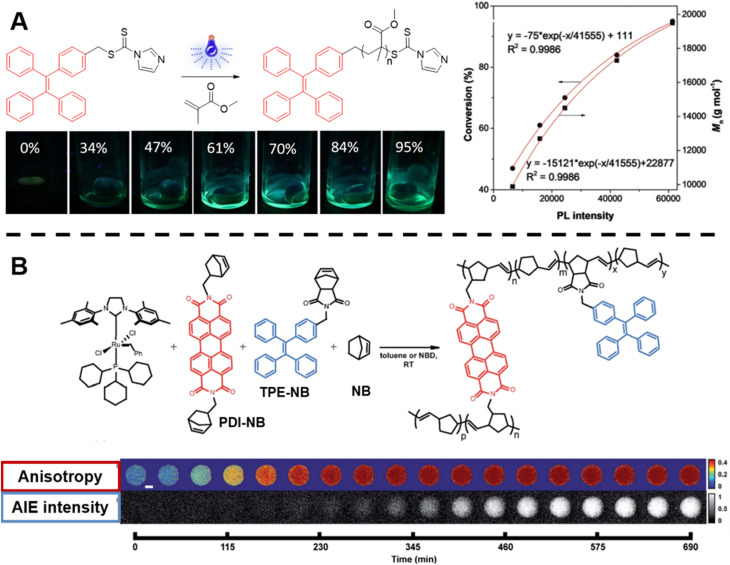
(A) Reaction scheme of photo-controlled RAFT polymerisation of MMA using TPE-containing dithiocarbamate as a RAFT agent, series of photographs showing the increase of fluorescence intensity with conversion of reaction and plot of conversion and molecular weight against light intensity. Reproduced from ref. 135 with permission from Wiley, copyright 2018. (B) Scheme of ROMP polymerisation with TPE- and PDI-labelled norbornene monomers as fluorescent probes (top) and visualisation of the polymerisation process over time based on AIE-Intensity and anisotropy signals (bottom). Reproduced from ref. 137 with permission from The Royal Society of Chemistry, copyright 2020.

In photo-controlled RAFT polymerisation, Yeow *et al.* utilised 5,10,15,20-tetraphenyl-21*H*,23*H*-porphine zinc (ZnTPP) as a photocatalyst and fluorescence probe to mediate polymerisation and report on monomer conversion.^136^ The fluorescence change of the system is likely due to the incorporation of ZnTPP into the polymer chain. Likewise, to visualise RAFT polymerisation *in situ*, Liu *et al.* developed an approach based on AIE (Fig. 3A).^135^ AIE describes the opposite of aggregation-caused quenching (ACQ), which is a common phenomenon when dyes aggregate. The fluorophores used in AIE, known as AIEgens, often show strong emission in the aggregated state due to the restriction of intramolecular motion, which makes them good candidates for sensing viscosity or other environmental changes.^75^ In their work, tetraphenylethylene (TPE)-containing dithiocarbamates were designed and synthesised. These compounds play the role of RAFT agents for the control of the polymerisation process. Meanwhile, TPE (a typical AIEgen) can be incorporated into the polymer chain upon light irradiation and thus sense the viscosity change during polymerisation. As noted, AIE may be more suitable for polymer systems with relatively large molecular weights.

In order to track polymerisation reactions on extended time scales, Cavell *et al.* combined two optically orthogonal readouts, AIE intensity and fluorescence polarisation anisotropy, by incorporating TPE- and perylene diimide (PDI)-labelled monomers (very low amounts, ppm and even below) into the ROMP of norbornene within a single microdroplet (Fig. 3B, top).^137^ Fluorescence polarisation anisotropy can quantify the rotational time scale of a fluorescent molecule, further providing information about the chemical evolution of its environment. As the polymerisation proceeds, anisotropy increases due to the loss of rotational freedom upon the monomers' incorporation into the growing polymer chain. Anisotropy is sensitive at early temporal regimes of the reaction, thus together with AIE can provide complementary information (Fig. 3B, bottom).

Here, ROMP is one type of controlled polymerisation that converts cyclic olefins into a polymer material. The key feature of ROMP is that any monomer-associated unsaturation is retained during the conversion of monomers into polymers, which allows ROMP to synthesise polymers with unique architectures and useful functions.^28^

**Fig. 4:**
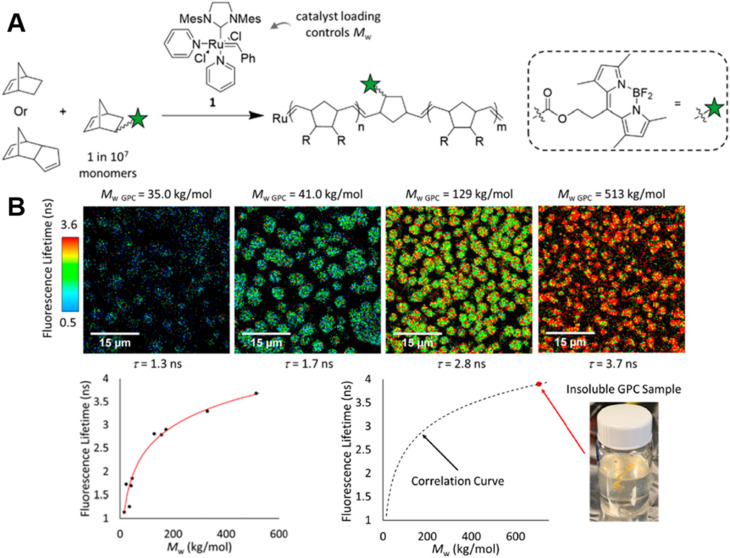
(A) ROMP polymerisation of dicyclopentadiene (DCPD) containing a small amount of BODIPY-labelled monomers. (B) FLIM images showing the increase in fluorescence lifetime with higher molecular weight during the ROMP reaction (top), plotted correlation between fluorescence lifetime and molecular weight and extrapolation of the molecular weight of insoluble material from the correlation curve (bottom). Reproduced from ref. 85 with permission from The American Chemical Society, copyright 2022.

In order to monitor ROMP, Iv *et al.* doped the initial reaction solution with a very small amount of fluorophore-labelled norbornene monomers, but, in this case, with BODIPY-labelled monomers (Fig. 4A) and applied advanced fluorescence microscopy – FLIM.^85^ As mentioned earlier, fluorescence-based techniques have significant advantages over conventional techniques: in addition to real-time characterisation, they may allow for determination of monomer conversions and molecular weights of insoluble polymers. Their work effectively demonstrates these advantages. The correlation between polymer fluorescence lifetime measured by FLIM and molecular weight allows readout of the molecular weight of polydicyclopentadiene (polyDCPD) in ongoing reactions, as well as molecular weight calculations for GPC-solvent insoluble, high-molecular weight, cross-linked polyDCPDs (Fig. 4B). Not only that, FLIM is capable of providing spatiotemporally resolved information on polymer morphology, which plays an important role in the properties of polymers, and the use of fluorescence techniques for morphology studies will be discussed in later sections.

Another advantage of fluorescence-based techniques is their ability to determine molecular weights of conjugated polymers, which are often overestimated by GPC. Tian *et al.* employed single-molecule fluorescence spectroscopy to determine the molecular weight of conjugated polymers using a single-molecule counting method.^138^ However, spin-casting of the polymer matrix in which these polymers are embedded is required. In order to count molecules in a solution phase, fluorescence correlation spectroscopy (FCS),^139,140^ a powerful tool for detecting molecular dynamics, may be a good choice.

Notably, the aforementioned fluorophores, especially viscosity/rigidity-sensitive fluorophores, typically blueshift their emission or merely alter fluorescence intensities with the growth of polymer chains. To address the challenge of developing fluorophore probes with bathochromic emission shifts, Feng *et al.* developed phenanthridine-fused tri-azatruxene fluorophores (PTFs, chemical structure refer to Fig. 2) by judiciously selecting each unit in the fluorophore: (i) phenanthridine units to enhance the fluorophore/polymer interaction; (ii) a rigid planar conformation by fusing π-rings to suppress nonradiative decay; (iii) large π systems to promote electronic coupling and frontier molecular orbital energy level alignment between the fluorophore and the polymer.^141^ Finally, in the polymerisation of pentafluorophenylacrylate (PFPA), a significant fluorescent colour change from blue to red-orange with increasing polymer molecular weight was observed (Fig. 2), which can be ascribed to dipole–dipole and polar–π interactions between PTFs and polymeric matrixes to form charge-transfer complexes.

**Fig. 5:**
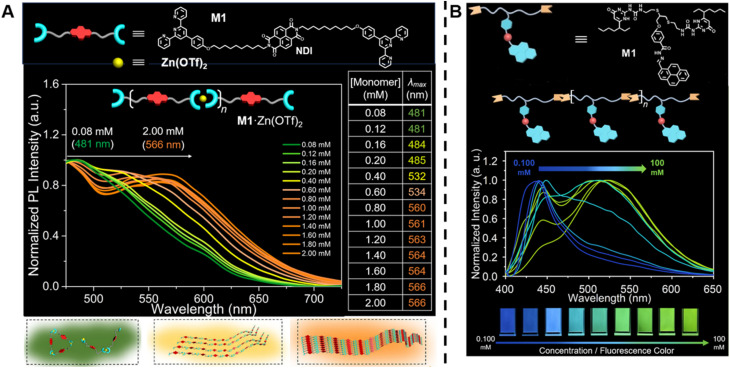
(A) Chemical structure of supramolecular monomers (top), plot of fluorescence emission spectra at different monomer concentration (middle) and scheme of supramolecular polymerisation process with bathochromic shifted fluorescent colour (bottom). Reproduced from ref. 152 (https://doi.org/10.1073/pnas.2121746119), under the terms of the CC BY-NC-ND 4.0 license [https://creativecommons.org/licenses/by-nc-nd/4.0/]. (B) Chemical structure of supramolecular monomers and schematic representation of supramolecular polymer formed by hydrogen bonding (top), plot of fluorescence emission spectra for increasing monomer concentration (middle) and visualisation of the fluorescent colour change (bottom). Reproduced from ref. 153 with permission from Wiley, copyright 2022.

In contrast to conventional covalently bound polymers, supramolecular polymers are polymeric arrays of repeat units that are connected together by highly directional non-covalent interactions,^36,142–144^ such as hydrogen bonds, π–π stacking and metal coordination.^6,142,143,145–151^ Due to these non-covalent interactions, supramolecular polymers are endowed with some special properties, including reversibility, recyclability and stimuli responsiveness, which can facilitate the design of responsive, self-healing and environmentally friendly materials.^6,36^ In order to determine molecular weight of a supramolecular polymer by optical methods, Sessler, Zhu, Ji and others proposed to incorporate two types of fluorophores into supramolecular polymer monomers, a J-type dye (its aggregates exhibit bathochromically shifted absorption bands) naphthalene diimide (NDI) (Fig. 5A, top) and an AIEgen pyrene benzohydrazonate-based fluorophore (Fig. 5B, top), respectively.^152,153^ The former monomers interact with Zn(OTf)_2_ to form supramolecular polymers through terpyridine-Zn^2+^ coordination (Fig. 5A, top). With increasing molecular weight resulting from the increasing monomer concentration, a change in the fluorescent colour from green to yellow to orange was observed (Fig. 5A, middle). This can be ascribed to the supramolecular assembly-induced aggregation of NDI groups, *i.e.*, the supramolecular polymerisation and polymer assembly process leads to dimeric forms of NDI followed by the formation of J-aggregates (Fig. 5A, bottom). Differently, the latter monomers polymerise through hydrogen bonding (Fig. 5B, top) and the molecular weight was visualised through aggregation-induced emission, *i.e.*, with the increase of molecular weight, a change of the fluorescent colour from dark blue to yellow-green was found (Fig. 5B, bottom). Polymer assembly will continue to be discussed in Section 5.

This section focuses on the principles of emission changes of various fluorophores, including fluorogenic molecules, AIEgens and molecular rotors, as well as their adaptability to the polymerisation process. Although NMR and SEC are the most common techniques for determining monomer conversion and molecular weight, fluorescence-based techniques are promising for *in situ* and real-time characterisation. Currently, conventional fluorescence spectrometry is the main adopted fluorescence technique, while – notably – advanced fluorescence techniques are able to access more types of polymers (*e.g.*, insoluble and conjugated polymers) and to provide more useful information, for example, FLIM for spatiotemporal morphological information and FCS for information about diffusion linked to polymerisation kinetics. Monitoring polymerisation processes from early to final stages and gaining deeper insights into polymerisation kinetics and mechanisms will aid in controlling polymerisation to obtain polymers with tailored properties and fluorescence-based techniques can critically assist along the way towards these goals.

## Polymer architecture

Inspired by nature's complexity, polymer scientists have made remarkable efforts in exploring synthetic polymers with sophisticated structures. Advanced polymerisation techniques, including living anionic polymerisation and RDRP, in combination with efficient linking reactions, such as click chemistry,^154^ have critically advanced the complexity and functionality of polymer architectures.^155–162^ Various well-defined architectures have been explored, including those of multiblock, star, comb/graft, cyclic, branched, dendritic, and network polymers.^161–170^ These architectures endow polymers with unique thermal (crystallinity, glass transition temperature), mechanical (density, viscosity, elasticity) and morphological properties, associating them with a plethora of applications, such as viscosity modifiers, dispersion stabilisers as well as nanomaterials for lithography and drug delivery.^166,169,171^ For example, dendrimer-like polymers have fascinating properties, including low intrinsic viscosity and intramolecular topological cavities, rendering such materials potential candidates for sensor and drug delivery applications.^172,173^

With flourishing research on synthesis and application of polymers with complex architectures, advanced analytical methods are needed for the characterisation of this complexity, which is essential for the synthesis and the study of properties of these polymers. Conventional methods, including rheometry, SEC and NMR, show significant drawbacks, especially for complex macromolecular architectures, for example in the quantification of crosslinking points in a network and calculation of arm length in multi-arm star polymers. Taking NMR as an example, the analysis suffers from signal overlaps from different groups. The overlap problem is severe for linear polymers due to broader absorption from similar groups, and becomes worse for more complex polymers. Thus, a direct, facile and robust platform is required to gain quantitative information of polymer architectures, for which fluorescence-based techniques are well suited.

**Scheme 1:**
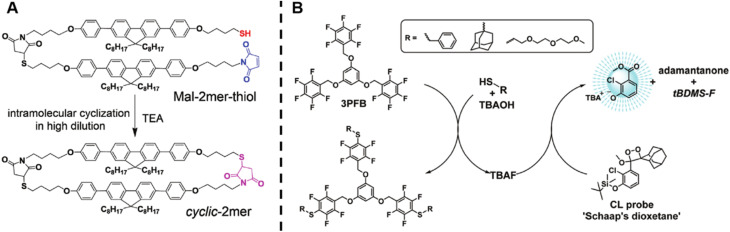
(A) Intramolecular thiol–maleimide cyclisation reaction. The consumption of the maleimide restores the fluorescence of the molecule and allows to monitor the reaction. Reproduced from ref. 174 with permission from The Royal Society of Chemistry, copyright 2017. (B) The PFTR between a linker (3PFB), a base (TBAOH) and a thiol, triggering the CL of Schaap's dioxetane. Reproduced from ref. 179 with permission from The Royal Society of Chemistry, copyright 2020.

Wang *et al.* successfully synthesised cyclic oligomers based on the intramolecular thiol–maleimide Michael addition reaction and monitored these cyclisation processes *in situ*.^174^ They first synthesised a dimer containing a fluorene moiety as a fluorescent group, a maleimide group and a thiol group, based on the thiol–maleimide Michael addition reaction (Scheme 1A). In this dimer, the fluorescence of fluorene is effectively quenched due to photo-induced electron transfer between fluorophores and adjacent electron-deficient double bonds of the maleimide group. The cyclisation reaction restores the fluorescence emission of fluorene, which increases with the consumption of maleimide groups. Furthermore, they successfully expanded the cyclic topology to a cyclic-brush-like topology by reducing cyclic oligomers, then grafting linear thiol-terminated polymers onto the backbone of cyclic oligomers. The degree of grafting can be monitored in a similar way. This work demonstrates the potential of maleimide-based fluorogenic probes as an effective method for monitoring topology formation. Here, it is worth mentioning that the thiol-Michael addition reaction, including the thiol–maleimide Michael addition, as a type of click reaction, is a powerful and widely used tool for the generation of polymers, even with complex architectures.^175^ Besides, the maleimide group is a robust group involved in a plethora of reactions.

The *para*-fluoro-thiol reaction (PFTR), another member of the click reaction family, also play an important role in polymer chemistry.^176–178^ Very recently, our group applied this reaction for the synthesis of three-arm star polymers by employing a trifunctional pentafluorobenzyl (3PFB) linker along with a poly(ethylene glycol) bearing a thiol end-group, and determined the number of PFTR events forming the polymer arm using a chemiluminescence (CL) read out (Scheme 1B).^179^ In PFTR, a thiol is deprotonated by a base and subsequently reacts with the *para*-carbon of a PFB moiety, releasing a fluoride ion. The fluoride ion can trigger the CL of Schaap's dioxetane^180^ and can thus serve as a quantitative read-out method. The large advantage of the CL method is that it requires neither an external light source, nor decomposition of polymer arms, therefore allowing easy access to star polymers as well as to more complex systems, as will be discussed later.

As one of the most studied polymer architectures, star polymers generally have compact structures and high segment density, making them excellent candidates for storing and delivering drugs.^166,181^ Among star polymers, miktoarm stars enable forming special morphological structures and supramolecular assemblies due to their chemical asymmetry, and thus have recently caught much attention.^167,169,182,183^ A crucial issue for miktoarm stars is the ratio/concentrations of different arm components, yet it is challenging to characterise them.

**Fig. 6:**
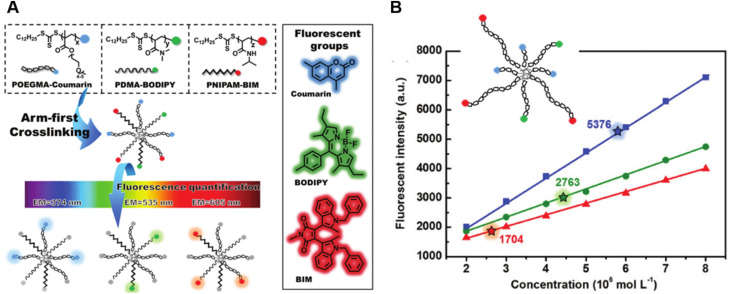
(A) Illustration of the arm-first approach for the synthesis of miktoarm star polymers bearing different fluorescent end-groups. (B) Linear fits of fluorescence intensities of mikto-star arms with the same monomeric units but different molecular weights, as shown in the inset, to quantify miktoarm star composition. Reproduced from ref. 184 with permission from The Royal Society of Chemistry, copyright 2022.

Very recently, Shi *et al.* introduced a fluorescence strategy for the direct quantification of arm species in miktoarm stars.^184^ A series of miktoarm star copolymers end-labelled with various fluorophores, including coumarin, BODIPY and bisindolylmaleimide (BIM), were prepared *via* an “arm-first” approach, in which the arm polymers were synthesised first by RAFT polymerisation, followed by the preparation of stars with the aid of a cross-linker (Fig. 6A). Due to the judicious choice of fluorophores, the concentrations and ratios of different arm components in these stars were successfully quantified by measuring fluorescence emission intensities of different fluorophores. These results reveal the ability of arm polymers with different monomeric units and molecular weight to form stars. To further demonstrate the capability of this fluorescence strategy, they applied it to quantify miktoarm stars bearing arms of the same monomeric units but different molecular weights, which cannot be characterised by NMR techniques (Fig. 6B).

Polymer networks, referring to polymer molecules with a high degree of crosslinking, represent another very important category of polymer architectures. They are of considerable interest for applications such as tissue engineering, separation, self-healing and lithography.^185–189^ Controlling the molecular structure of networks is critical for tailoring chemical and mechanical properties to these applications. The advent of controlled polymerisation methods and highly efficient linking reactions, *e.g.*, click reactions, has led to a significant progress in the synthesis of better-defined polymer networks.^161,190^ However, detailed characterisation of networks including the number of crosslinks and structural defects remains challenging.

**Fig. 7:**
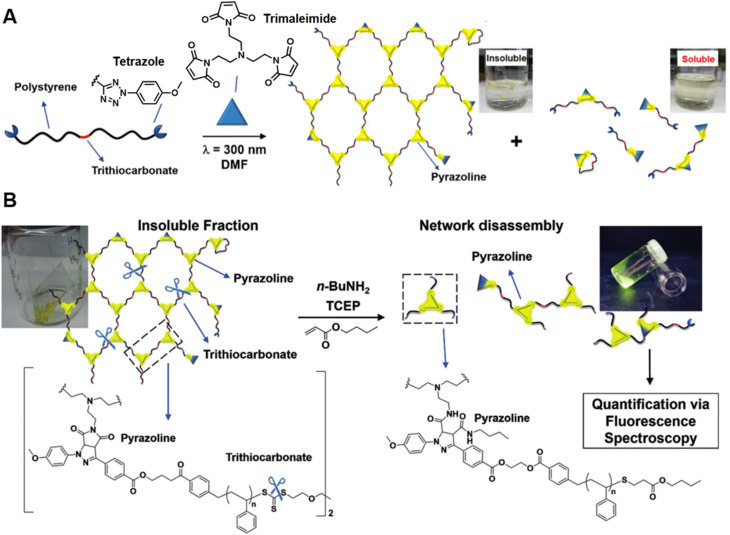
(A) UV-induced NITEC crosslinking reaction between tetrazole chain-ends and trimaleimide cross-linkers, forming insoluble and soluble fractions. (B) Network disassembly *via* aminolysis. Determining the concentration of pyrazoline after aminolysis *via* fluorescence spectroscopy allows to quantify the number crosslinking points within the former network. Reproduced from ref. 191 with permission from Wiley, copyright 2018.

Our group has successfully synthesised well-defined polymer networks by combing RAFT polymerisation and the NITEC reaction (mentioned in Section 2) and introduced a powerful fluorescence-based methodology to quantify the number of crosslinking points in the resulting networks.^191^ RAFT polymerisation allows for the incorporation of pro-fluorescent tetrazole moieties at the termini of polystyrene (PS) chains, and their subsequent reaction with trimaleimide *via* NITEC forms networks with a fluorescent pyrazoline ring at each linkage point (Fig. 7A). After network disassembly (*i.e.*, cleavage) *via* aminolysis of the trithiocarbonate moieties, all fluorescent crosslinking points are preserved in a soluble system and the number of crosslinking points becomes quantifiable *via* fluorescence-readout, based on the correlation between pyrazoline concentration and fluorescence intensity (Fig. 7B). Our method is in-principle applicable to all polymers capable of RAFT polymerisation. In addition, photoactivated cycloaddition NITEC offers spatiotemporal control over the crosslinking process. To avoid network degradation, a chemiluminescence read-out method to assess network formation in PFTR systems was proposed by our group,^179^ which was carried out similarly to the characterisation of arm formation in star polymers described above.

Mimicking nature's complexity has given rise to polymers with sophisticated architectures like star, graft and multiblock polymers. Currently, the scientific community is developing effective methods to generate polymers with more complex architectures and controllable dispersity. To complement the increasing complexity of polymer architecture, advanced analytical methods are required to study the synthesis and properties of these polymers. Despite the small number of examples, fluorescence-based techniques have, by providing information on miktoarm star copolymers and polymer networks, well demonstrated their ability to analyse polymer architectures. We anticipate that exploiting more fluorescence principles (*e.g.*, FRET) and advanced techniques (*e.g.*, single-molecule fluorescence microscopy) will give deeper insights into polymeric architectures.

## Polymer conformation

Polymer conformation describes the three-dimensional arrangement of the constitutive atomic groups of a polymer. It is perhaps the most important concept in polymer physics, as it has a profound influence on the polymer's physical properties. For instance, polymer diffusion – a process relevant to a plethora of applications of polymers, including polymeric membranes, drug delivery and self-healing^192,193^ – is a direct consequence of conformational changes.^194–196^ The various conformations of a polymer chain are a consequence of rotations around single bonds within the polymer backbone, depending on chain flexibility and interactions between monomeric units as well as with surroundings (*e.g.*, solvent).^197^ Given the immense number of conformations and multiplicity of interactions, various models and theories have emerged to simplify the study of polymer conformations, such as the worm-like chain model for describing semiflexible polymers and the Flory theory for determining the size of a polymer by considering excluded volume interactions and elastic entropy.^198–202^ The important parameters in the study of polymer conformations include the end–end distance of the polymer chain (*R*_ee_), the radius of gyration (*R*_g_) defined as the root of the mean-square distance of a monomer from the centre of mass of the chain averaged over all monomers,^203^ and their respective distributions.

Correspondingly, experimental characterisation and data acquisition is of considerable importance to validate and improve different models and theories which describe polymer conformations. At present, scattering techniques, including light scattering, X-ray scattering and neutron scattering, are the most widely employed techniques.^204–208^ However, light scattering can only provide size information for polymers with very high molecular weights or polymer ensembles due to the relatively large wavelength of light. X-ray and neutron scattering allow smaller detection scales, but specialised equipment and/or chemistries are required, such as synchrotron light sources for small-angle X-ray scattering as well as spallation sources and deuteration for neutron scattering.^209^ In addition, all scattering experiments provide reciprocal space data and must be paired with complex fitting models to obtain the size of polymer chains.

Alternatively, fluorescence-based approaches may allow the facile and direct measurement of polymer conformations. They offer advantages including fast data acquisition, *in situ* measurement as well as labelling and location specificity. Among various fluorescence phenomena, excimer formation and FRET are particularly useful to study polymer chain conformations.^210–215^ In both cases, the initial step is to label individual polymer chains at specific positions.

An excimer, *e.g.*, an excited pyrene dimer (as mentioned before), is formed by collisional association of a ground state molecule with an excited one. Time-resolved fluorescence measurements show that only emission of pyrene in the end-labelled monodispersed polymers is observed immediately after excitation. The broad and structureless emission then grows as excimers are formed by diffusional encounters of the excited pyrene covalently attached to one end of a polymer chain and the ground state pyrene at the other chain end.^211,216^ The average translational diffusion coefficient (*D*_trans_) of polymer chains within a certain molecular weight can thus be determined *via* the intramolecular quenching rate constant *k* calculated from the fluorescence decay curve.^211^

**Fig. 8:**
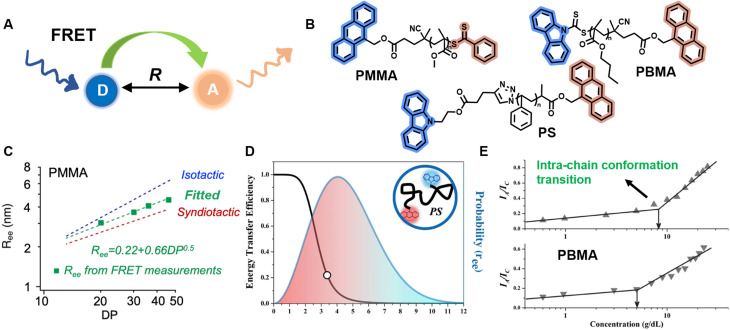
(A) Schematic illustration of the FRET mechanism. (B) Chemical structures of three FRET donor–acceptor end-labelled polymers from ref. 214, 212 and 223 synthesised by ATRP or RAFT polymerisation. (C) Plot of *R*_ee_ of PMMA determined *via* FRET as a function of degree of polymerisation, corresponding to simulated ones. Reproduced from ref. 214 with permission from The American Chemical Society, copyright 2023. (D) FRET efficiency against *R*_ee_ (black curve) and probability distribution of *R*_ee_ (blue curve) of PS. Reproduced from ref. 212 with permission from The American Chemical Society, copyright 2016. (E) Dependence of the ratio of fluorescence intensities between FRET acceptor and donor on the concentration of PBMA. The marked sharp increase indicates the intra-chain conformation transition. Reproduced from ref. 223 with permission from Wiley, copyright 2016.

As a comparison, methods based on FRET can provide further information about polymer conformation, such as *R*_ee_, with a larger *R*_ee_ indicating a more extended conformation.^212,214^ FRET describes non-radiative energy transfer occurring *via* dipole–dipole coupling from an excited state fluorophore (donor, D) to a ground state molecule (acceptor, A) under the condition of sufficient spectral overlap between the donor emission and acceptor absorption and close proximity between donor and acceptor (Fig. 8A).^72^ Due to the inherent inverse-sixth-power distance dependence of FRET efficiency, it enables nanoscale proximity detection with a typical range of 1–10 nm.^72,73^ The efficiency can be determined by measurement of fluorescence intensity or lifetime.

FRET as a molecular scale ruler has been used extensively to obtain quantitative conformational information in bio-related systems, such as peptides, DNA and proteins.^210,217–220^ Controlled polymerisations and click chemistry have significantly advanced the synthesis of well-defined polymer structures containing fluorescent end-groups,^212,215,221^ which brings unprecedented opportunities for FRET methods to quantify synthetic polymer conformations, unlike previous qualitative analyses.^212,215,222,223^

Sha *et al.* prepared heterotelechelic PS and PMMA possessing FRET pairs (carbazole as donor and anthracene as acceptor) at chain ends through the combination of ATRP and click chemistry or RAFT polymerisation alone using a di-functionalised RAFT agent (Fig. 8B).^212,223^ In another work, Simon, Qiang and others also applied RAFT polymerisation for the synthesis of FRET paired-end labelled PS and PMMA, but with anthracene as donor and the phenylthiocarbonylthio part as acceptor (Fig. 8B).^214^ These polymers have controlled molecular weight, low dispersity and nearly stoichiometric amounts of donor and acceptor molecules, thus enabling the determination of average *R*_ee_ of polymer chains and their distribution in solution through FRET efficiency, which can be calculated through the relative emission intensities of the donor and acceptor measured by fluorescence spectroscopy. Notably, in the work of Simon and Qiang, the average *R*_ee_ determined through the FRET measurements is in agreement with simulation results obtained from all-atom molecular dynamics (Fig. 8C); the distribution of *R*_ee_ in Sha's work obeys Gaussian statistics (Fig. 8D).

Solution concentration has a significant effect on polymer chain conformation. According to the scaling theory of polymer solutions,^224^ in diluted solution, polymer chains are isolated and behave as single coils. With increasing concentration, the distance between these coils becomes smaller. At the critical concentration (c*), the polymer coils start to come into contact with each other, causing them to assume a more densely packed state. Following that, the coils overlap and interpenetrate, and the solution is termed as semidilute. Finally, polymer chains are strongly entangled at concentrated solutions. The importance of c* in the study of polymer solutions cannot be overstated, as the chain overlap above c* results in a new conformational scaling behaviour.^224,225^ FRET has been employed to study the inter- and intra-chain conformation transition and determine c* (Fig. 8E).^213,223,226^ Moreover, it also represents an efficient and simple tool to determine the influence of temperature, solvent polarity and viscosity on polymer conformational changes.^215,227^

Despite the significant potential of FRET spectroscopy in studying polymer chain conformations, several problems may be encountered. For instance, diffusion-enhanced FRET efficiency^228^ and interchain FRET may disturb the measurements of *R*_ee_, and the applicable distance scale of FRET limits conformational studies of long polymer chains.

SMLM constitutes a very attractive alternative for the study of polymer conformation by locating the exact positions of fluorescent labels in a single polymer chain.^229–232^ It can reveal rare events, allows access to distributions and time- and space-dependent properties, and helps understanding how molecular-scale behaviour gives rise to bulk (macroscopic) properties, while ensemble measurements, *e.g.*, FRET-based fluorescence spectroscopy, yield information only on average properties. Furthermore, fluorescence microscopy is capable of imaging polymer chains in their native environment, which is challenging for other microscopy techniques, such as AFM and TEM. AFM cannot probe polymers in their native environment due to the lack of sufficient contrast between polymer chains, while TEM requires invasive sample preparation and high-vacuum environments.

Revealing single polymer chains through SMLM relies on the deactivation of fluorophores during image acquisition.^233,234^ Fluorophores labelled onto a polymer chain are typically a few nm in size, but, due to the optical diffraction limit, they appear as Airy discs of 200–300 nm diameter under optical microscopy.^235^ Even so, single, isolated emitters can be localised with nanometre precision by fitting their spot images with point-spread functions (PSFs, *e.g.*, 2D Gaussian function).^236^ Thus, one can construct an image of a polymer chain with nanometre resolution and calculate the distance between labels once the positions of all the emitters in a polymer chain are determined. However, the real distance between two positions on the chain is generally smaller than the optical resolution and thus the PSFs of different fluorophores will blur together and cannot be individually resolved. SMLM usually exploits the fact that fluorophores stochastically switch between an active ‘ON’ or ‘bright’ state, where they emit fluorescent light, and one or more inactive ‘OFF’ or ‘dark’ states in which they do not emit light.^69–71,237,238^ Under suitable conditions, only a small fraction of these molecules is ‘ON’. The OFF/ON/OFF switching leads to fluorophore ‘blinking’, their PSFs can thus be spatially separated and be fitted to determine their positions. By acquiring thousands of widefield image frames and subsequent computational processing of these images, the coordinates of a sufficient number of fluorophores can be determined and accumulated to generate a pointillistic image. Depending on the experimental parameters, images with features as small as a few nm can be achieved.^233,234^

Compared to studies of biopolymers, SMLM for the study of synthetic polymers is still in its infancy, but it undeniably has enormous potential for revealing the properties of synthetic polymers, including their conformations.

Aoki *et al.* have determined the *R*_ee_ of individual PMMA chains in an ultra-thin film and in a bulk state and quantitatively examined the distribution of *R*_ee_.^230^ In their system, both ends of PMMA were labelled with PDI. At the beginning, both PDIs emitted the fluorescence, but after a while one of them was photo-bleached and only the emission from the other was observed. Fitting the PSF from one emitter and subsequently from both allows the evaluation of *R*_ee._ It is worth noting that the efficiency of these measurements is low, as only a very low number of chains exhibit such emission behaviour.

**Fig. 9:**
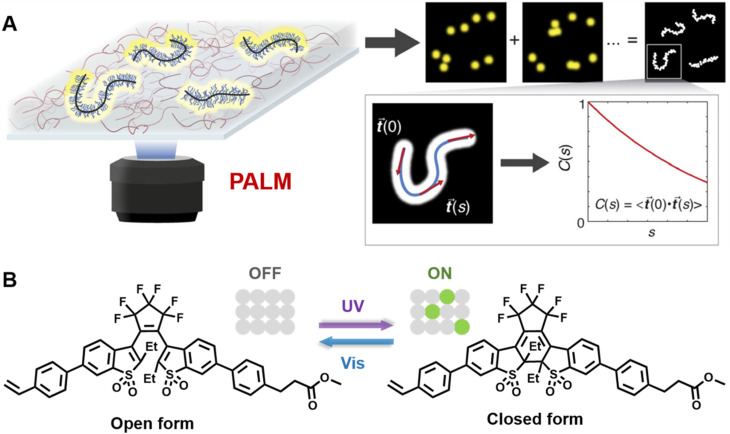
(A) Illustration of PALM SMLM for the imaging of individual bottlebrush polymers. Repeated, stochastical activation of a small fraction of fluorophores that are attached to the polymer allows localisation of each fluorophore by fitting to the PSF. Images of the polymers can thus be reconstructed and allow analysis of chain conformation (persistent length). Reproduced from ref. 239 (https://www.pnas.org/doi/abs/10.1073/pnas.2109534118), under the terms of the PNAS License to Publish, copyright 2021. (B) Chemical structure of the diarylethene derivative employed for PALM imaging in ref. 239 and schematic of the photoswitch between open (‘OFF’) and closed (‘ON’) form.

Very recently, Wang and others provided clear images of individual bottlebrush polymer chains in a polymer melt composed of linear polymers and studied the dependence of conformation (here, the persistent length, quantifying the rigidity of a polymer) on the molecular architecture (*i.e.*, side chain length and grafting density) and the features of the surrounding environment (Fig. 9A).^239^ Photoswitchable diarylethenes were selected to label the polymer chains. Under UV light irradiation, diarylethenes can be activated to an ‘ON’ state (closed form), which can convert back to the ‘OFF’ state (open form) by blue light (Fig. 9B).^240^ Diarylethene-labelled polymer chains combined with two wavelengths enable the use of photo-activated localisation microscopy (PALM) – one of the SMLM techniques – to study polymer conformation.

Characterisation of polymer conformation is of great importance for understanding the physical properties of polymers. FRET as a molecular scale ruler has been established in fluorescence spectroscopy (or microscopy) to provide quantitative conformational information. Single-molecule fluorescence microscopy provides intriguing opportunities to study individual polymer chain conformation. It can reveal not only the distance between two locations, but also orientation and conformation dynamics, even in 3D.^231,241,242^ The non-invasive characteristic of fluorescence-based techniques allows the characterisation of polymer conformation in their native environment and thus gain a better understanding of conformational behaviours. More effort can be devoted to polymer chains with complex architectures in addition to linear chains.

## Polymer self-assembly

Inspired by the self-assembly of biopolymers, *e.g.*, peptides and proteins, the self-assembly of block copolymers, consisting of two or more chemically distinct building blocks, represents an important method for the preparation of morphologically diverse nano-objects, which are highly promising in a broad range of applications, including biomedicine, catalysis, energy storage, lithography and devices.^243–249^ A precisely designed block copolymer architecture is a key prerequisite for controlling self-assembly processes. Controlled polymerisation techniques, including living anionic polymerisation and RDRP, in combination with various linking reactions, offer exceptional opportunities to access a variety of well-defined block copolymers, including linear, star, graft and cyclic architectures, greatly enriching the chemistry and functionality of self-assembled systems.^250–254^ Self-assembly of block copolymers can be conducted in bulk or in solution. Bulk self-assembly is usually induced by thermal or solvent annealing, where microphase separation, driven by thermodynamic incompatibilities between polymers, forms a wide range of nanostructures, such as spheres, cylinders, lamellae and gyroids.^255–259^ The morphology of the resulting structures depends on a variety of parameters, including the volume friction of blocks, the degree of polymerisation and the Flory–Huggins interaction parameters.^255,256^ Solution self-assembly of block copolymers is a complex process governed by the interactions of polymer segments with the solvent as well as with each other. Owing to the distinct solubility of different polymer segments in selected solvents, block copolymers can self-assemble into various morphologies, including spherical micelles, cylinders (or worms or rods) and vesicles.^260–263^ More complex morphologies, such as multicompartment assemblies, can be achieved by tuning polymer composition, solvent composition and temperature, as well as by applying crystallisation or noncovalent supramolecular interactions, including electrostatic interactions, metal coordination, hydrogen bonding and hydrophobic forces.^254,264–272^ In addition to developing more complex morphologies, a current focus of the scientific community is the precise control of polymer self-assembly.^6^ One feasible method to control self-assembly processes has been offered by Winnik, Manners and co-workers – living crystallisation-driven self-assembly (CDSA).^273–275^ In their system, for example, the addition of block copolymer unimers containing a crystallisable core-forming block to cylindrical seed micelles with crystalline cores can lead to micelle growth from the crystalline core termini, allowing access to monodisperse cylinders with lengths determined by the unimer-to-seed ratio. This process can be considered analogous to living covalent polymerisation, *e.g.*, RDRP.^273^ Solution self-assembly is usually carried out in a multi-step process and in dilute solutions, which limits the scaling-up of the preparation.^276^ PISA offers an attractive solution to overcome these limitations.^277–280^ It combines polymerisation and self-assembly and can be performed at high weight percentages of solids.^97,276,281^

Turning to supramolecular polymers, on the one hand, molecular building blocks can be polymerised *via* covalent synthesis into polymers with complex architectures, which then self-assemble into various morphologies.^254,270,282^ On the other hand, molecular building blocks can be non-covalently linked and further undergo self-assembly.^6,143,150,283^ As mentioned earlier, various non-covalent interactions can be employed to construct supramolecular polymers.^142,143,145–151^ The reversible nature of these interactions and the structure of the building blocks endow these polymers with unique properties, including the responsiveness to their surrounding environments.^6,284,285^ Supramolecular polymers are an integral part of the field of self-assembly and show potential applications in a range of area from electronics to biomedicine.^36,63,286–291^

Characterisation of the properties of self-assembled structures as well as of the polymer self-assembly process is essential to advance our fundamental understanding of polymer self-assembly and subsequently enables us to optimise performances of structures for desired applications.

Arising from its sensitivity, fluorescence spectroscopy has been exploited to provide a wealth of information about changes in the morphology of assemblies (*e.g.*, from assembly to disassembly, change of size) and properties of assemblies, such as their critical aggregation concentration (CAC, the concentration at which aggregates are formed) and the glass transition temperature (*T*_g_) of the core of micelles, based on changes in emission intensity, wavelength or lifetime *via* fluorescence quenching, FRET, AIE, *etc.*^250,251,292–296^

**Fig. 10:**
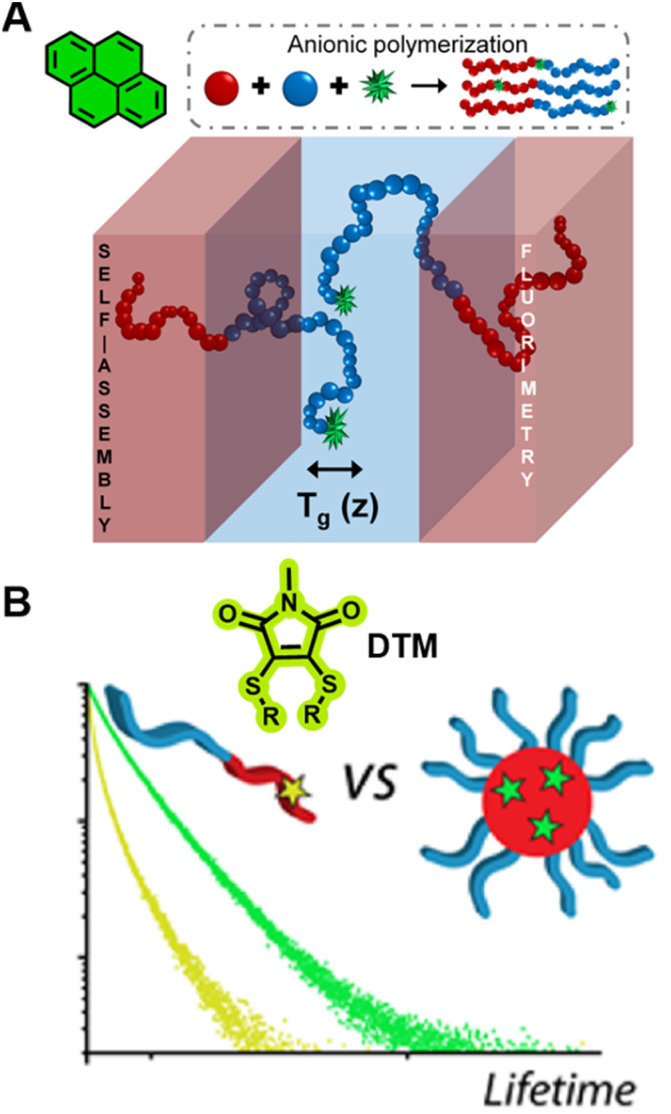
(A) Characterisation of *T*_g_ in the block copolymers self-assembled lamella *via* fluorometry, which is enabled by the precise location of pyrene in the block copolymer. Reproduced from ref. 298 with permission from The American Chemical Society, https://pubs.acs.org/doi/10.1021/acscentsci.8b00043, copyright 2018 (further permissions related to ref. 298 are to be directed to the ACS). (B) Chemical structure of DTM and comparison of the fluorescence lifetime of DTM in a unimer and in a self-assembled micelle. Reproduced from ref. 300 (https://doi.org/10.1021/acs.macromol.5b02152), under the terms of the CC BY 4.0 license [https://creativecommons.org/licenses/by/4.0/].

In bulk self-assembled lamella, a large fraction of polymer segments can be located within a few nanometres of an internal interface, a region with properties that can be strongly modified and significantly different from those of homogeneous and block polymers.^297^ Due to the strong sensitivity of pyrene's fluorescence intensity to the nanoscale medium, the characterisation of *T*_g_ over different length scales (nm) of the lamella can be achieved *via* fluorescence spectroscopy using fluorescent pyrene-bearing monomers precisely placed at specific locations along the polymer chain (Fig. 10A).^298^ The strong dependency of fluorescence intensity and lifetime of BODIPY-C12 on the viscosity of its surrounding environment allows multiparameter characterisation of self-assembled polymers, including the critical micelle concentration (CMC, concentration of block copolymers above which micelles form) and critical micelle temperature (CMT, the transition temperature, above or below which the formation of associated structures becomes appreciable) *via* steady-state fluorescence spectroscopy as well as micelle core phase transition temperatures, microviscosity within the micelles and the sol–gel transition temperature *via* time-resolved fluorescence spectroscopy.^299^ The use of molecular rotors can thus eliminate tedious multi-instrument processes for the characterisation of polymeric self-assembly.

Robin *et al.* have synthesised block copolymer micelles with a fluorescent dithiomaleimide (DTM) covalently linked to the micelle core or shell.^250,300^ The transition from micelle to unimer can be detected for the core-labelled micelles by fluorescence lifetime measurements, based on solvent collisional quenching, *i.e.*, a good protection of the DTM fluorophore from solvent quenching (*e.g.*, the DTM is located within the dehydrated core) results in a longer lifetime of the micelles (Fig. 10B). Furthermore, the core-labelled micelles can self-report on the presence of a fluorescent hydrophobic guest as a result of FRET between the DTM fluorophore and the guest.

FRET has been utilised as a powerful analytical tool to measure CACs and CMCs, to identify the stability of assemblies and morphological responses to stimuli, to follow the encapsulation and release of fluorescent payloads, as well as to probe the dynamic-exchange phenomenon in block copolymer assemblies and supramolecular polymers.^296,301–303^

**Fig. 11:**
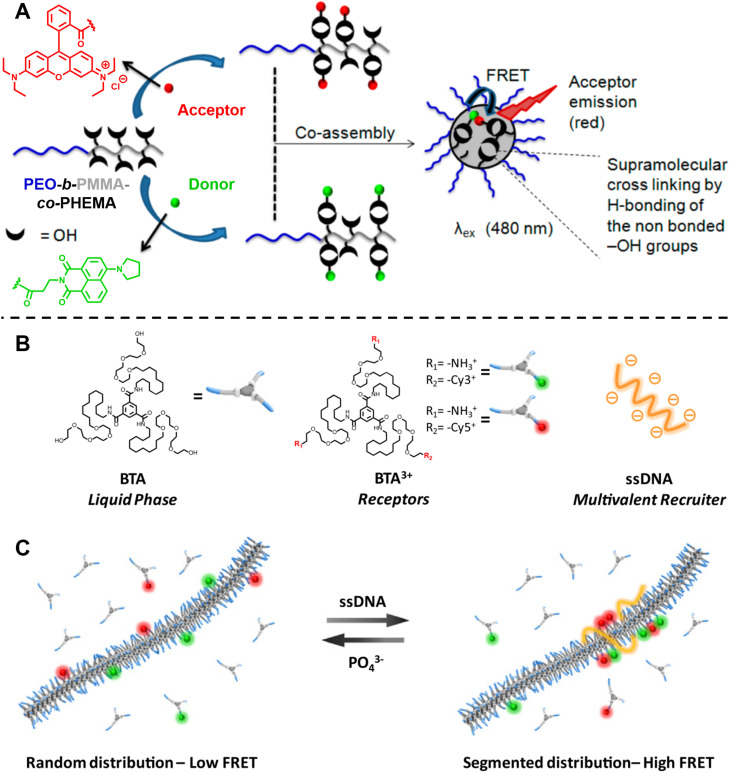
(A) Co-assembly of block copolymers with donor- or acceptor-labelled hydrophobic blocks into micelles. FRET allows to monitor micelle formation and exchange rate between the micelle and unimer. Reproduced from ref. 301 with permission from The American Chemical Society, copyright 2015. (B) Chemical structures of neutral monomers (BTA), cationic receptors labelled with Cy3 or Cy5 (BTA^3+^) and multivalent recruiter (ssDNA). (C) Schematic representation of the reversible clustering of receptors along the supramolecular polymer. A recruiter triggers clustering of receptors, leading to high FRET efficiencies. (B and C) Reproduced from ref. 82 (https://doi.org/10.1073/pnas.1303109110), under the terms of the PNAS License to Publish, copyright 2013.

Ghosh and colleagues synthesised block copolymers polyethylene glycol (PEO)-*b*-PMMA-*co*-polyhydroxyethylmethacrylate (PHEMA) with either green (donor, 1,8-naphthalimide derivative) or red (acceptor, rhodamine B) fluorescent dyes covalently attached to the hydrophobic blocks.^301^ Mixing the red- and green-labelled polymers results in highly efficient FRET due to the close proximity between the donor and acceptor after co-assembly of these polymers (Fig. 11A). The CAC, the dynamics of micellisation and the tolerance of the formed micelle aggregates to different solvents can also be investigated by the FRET technique. Besides, this technique allows to monitor the exchange rate between the micelle and unimer, aiding to understand the dynamics and stability of these micelles. Similarly, they also applied FRET to study the co-assembly and self-sorted assembly behaviour between different dye-labelled polymers in a molecular interaction-driven self-assembly system;^270^ Huang *et al.* employed FRET to determine the kinetics of coordination-driven self-assembly processes and to investigate the solvent and anion effect in self-assembly, the stability of metallosupramolecular structures and the dynamic ligand exchange between structures.^149^

Albertazzi *et al.* presented the synthesis of multicomponent one-dimensional supramolecular polymers *via* co-assembly of neutral 1,3,5-benzenetricarboxamide (BTA) monomers with cationic species BTA^3+^, labelled with FRET pairs (cyanine Cy3/Cy5) (Fig. 11B), and demonstrated that a multivalent recruiter (*e.g.*, ssDNA with multi-charged groups) is able to bind selectively to the BTA^3+^ monomers (as receptors) and trigger their clustering (Fig. 11C).^82^ The dynamics of supramolecular polymers synchronised with the multivalency of a binder can afford spatiotemporal control over the monomer sequence. Here, FRET has been exploited to evaluate the dynamics of the multicomponent supramolecular polymers, the clustering kinetics as well as the effect of recruiters and receptors on monomer clustering.^82,284^

**Fig. 12:**
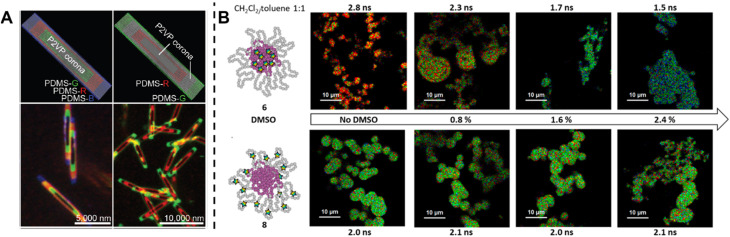
(A) Schematic representations and CLSM images of self-assembled platelet micelles, selectively functionalised with fluorescent labels. Reproduced from ref. 275 with permission from The American Association for the Advancement of Science, copyright 2016. (B) FLIM images showing block-selective solvation changes during gradual addition of DMSO to core and shell-labelled micelles in CH_2_Cl_2_/toluene. Reproduced from ref. 308 with permission from The American Chemical Society, copyright 2023.

Fluorescence spectroscopy provides in-depth information of self-assembled systems, but it is of high significance to enable optical imaging of these structures. Confocal microscopy, most frequently confocal laser scanning microscopy (CLSM),^304,305^ has been used extensively for visualisation and analysis of samples.^107,306,307^ The excitation light in confocal microscopy is focused through an objective lens to a diffraction limited spot. The generated fluorescence is collected by the same objective lens, passes through appropriate filters and pinholes blocking out-of-focus light, and finally reaches a point detector. The complete image is then created by scanning across the sample.^305^ Using living CDSA, Manners, Winnik and co-workers have prepared well-defined cylindrical multiblock micelles with distinct domains and rectangular platelet micelles with tuneable dimensions (Fig. 12A).^275,307^ Imaging of each colour by CLSM reveals the distribution of different domains, which are almost indistinguishable by electron microscopy. FLIM, another advanced imaging technique, bases its contrast on the fluorescence lifetimes (extrapolated from excited state decay curves) of a fluorescent sample, rendering this technique unaffected by the concentration of fluorophores, excitation intensity and photo-bleaching.^66,131^ The high spatial resolution of FLIM combined with the high sensitivity of covalently attached (separately labelling the shell and core) molecular rotors to their microenvironments has been used by Eivgi *et al.*, to reveal the behaviours and mechanisms of block-selective solvent-triggered assembly and disassembly.^308^ For example, FLIM reveals that DMSO – a solvent that causes disassembly of the copolymer in their system – influences exclusively the polar block (Fig. 12B). Understanding differential block–solvent interactions can further aid in finely tuning block copolymer self-assembly. Notably, FLIM shows superior sensitivity over ^1^H NMR spectroscopy, and fluorescence microscopy can reveal the properties of specific regions.

Optical diffraction limits the imaging resolution of fluorescence microscopy, while the emergence of super-resolution fluorescence microscopy (SRFM) techniques^67–71,309^ has overcome this limit and improved resolutions, allowing the visualisation of behaviour down to the scale of single molecules. SRFM techniques have been widely applied in biology,^310^ in contrast, its application in polymer science is still in its infancy, nevertheless, polymer scientists have gained new, important and fundamental insights through the implementation of SRFM techniques.^311,312^ The most well-known methods include structured illumination microscopy (SIM), STED and SMLM.

SRFM is able to reveal the morphology and determine the size of self-assembled structures.^90,107,313–315^ With multi-colour imaging, it can reveal different blocks or domains.^91,107,150,316,317^ Its non-invasive and non-destructive, characteristics allow imaging of structures in their native environment.^91,316–319^ Furthermore, the high spatiotemporal resolution enables to visualise structure formation processes, study dynamic behaviours and reveal mechanisms and driving forces behind self-assembly.^83,88,319,320^

**Fig. 13:**
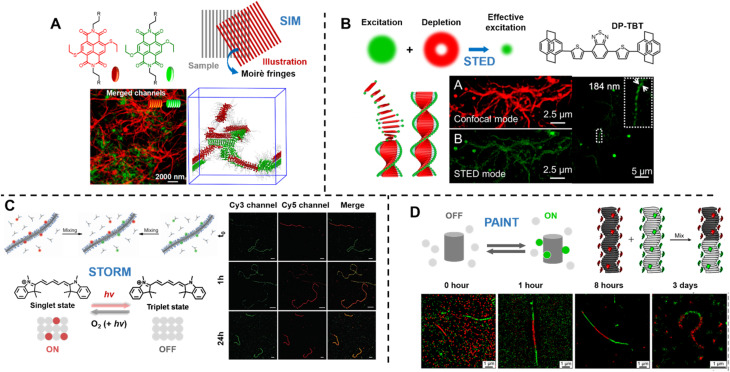
(A) Chemical structures of core-substituted naphthalene diimide π-conjugated monomers (top left), illustration of the SIM principle (top right), SIM image and schematic representation of self-sorted supramolecular polymers. Reproduced from ref. 150 with permission from The American Chemical Society, copyright 2020. (B) Mechanism of STED (top left), chemical structure of the AIEgen (DP-TBT) (top right), scheme of the helical self-assembly of DP-TBT and a side-by-side comparison of CLSM and STED images (bottom). Optimised STED can resolve individual turns of a helix (bottom right). Reproduced from ref. 318 with permission from The American Chemical Society, copyright 2019. (C) Illustration of the monomer exchange process of supramolecular fibres (top left), mechanism of STORM, upon irradiation a dye can enter a long-lived non-fluorescent triplet state (bottom left), STORM images of Cy3 and Cy5-labelled supramolecular fibres to follow monomer distribution at different times (right). Reproduced from ref. 83 with permission from The American Association for the Advancement of Science, copyright 2014. (D) Mechanism of PAINT, fluorescent dyes become visible when temporarily bound to a target structure (top left), illustration of copolymerisation of two supramolecular fibres bearing red- or green fluorescent labels (top right), iPAINT images at different times, visualising the dynamics of supramolecular fibres (bottom). Reproduced from ref. 319 (https://doi.org/10.1021/acsnano.8b00396), under the terms of the CC BY-NC-ND 4.0 license [https://creativecommons.org/licenses/by-nc-nd/4.0/].

Sarkar *et al.* introduced a two-component, sequence controlled, supramolecular copolymerisation to access self-sorted, random and block supramolecular polymers.^150^ The core-substituted naphthalene diimide π-conjugated monomers (Fig. 13A, top left) are not only capable of supramolecular polymerisation, but also allow the use of SIM to visualise the resulting multicomponent structures due to the orthogonal fluorescent nature (green or red emission) of monomers (Fig. 13A, bottom). SIM can surpass the optical diffraction limit by illuminating the sample with patterned light and subsequent software analysis to extract information from Moiré fringes (Fig. 13A, top right).^309,321,322^ It does not have specific requirements for fluorophores.^322^

STED and SMLM further break the diffraction limit by exploiting the photochemical and photophysical properties of fluorescent molecules.^67–71^

STED is related to conventional confocal microscopy, but uses a second beam to quench the fluorescence of undesired molecules to control the excitation volume and thus exceed the optical diffraction limit (Fig. 13B, top left).^323^ The second beam is usually a doughnut-shaped beam, whose wavelength is red-shifted relative to the excitation laser, and the excitation volume can generally be adjusted by tuning the depletion intensity. Dang *et al.* have used STED to visualise self-assembled helical fibres. These authors synthesised an AIEgen, DP-TBT (chemical structure refer to Fig. 13B, top right), which readily forms helixes (Fig. 13B, bottom).^318^ Meanwhile, DP-TBT exhibits highly emissive features in the aggregate state, a large Stokes shift and excellent photostability, meeting the stringent requirements for fluorophores in STED. DP-TBT-based self-assembled structures can thus be visualised by STED (Fig. 13B, bottom). Furthermore, long-time tracking using STED allows to monitor the formation and growth of helical fibres during the self-assembly process.

Manners' group applied STED and dual-colour SMLM to determine the lengths and length distributions of cylindrical micelles formed by living CDSA *in situ*.^91,106^ Importantly, both techniques enable the investigation of growth kinetics and the effects of various self-assembly parameters on the kinetics, providing guidelines for the future optimisation of a wide variety of living CDSA systems.

Molecular dynamic exchange is an important phenomenon in block copolymer assemblies and supramolecular polymers.^301,324–326^ FRET experiments can provide useful information about the time scale of exchange (see above), but are insufficient to unveil its mechanism.^82,83^ Moreover, these ensemble experiments fail to detect the diversity among self-assembled structures or within an individual structure.

Meijer's group has reported the use of stochastic optical reconstruction microscopy (STORM) to probe the dynamics of supramolecular fibres.^83^ Different dyes (Cy3 and Cy5) attached to BTA allow the use of multicolour STORM to determine the monomer distribution along the fibre backbone (Fig. 13C, top left). By following the monomer distribution during the molecular exchange process (Fig. 13C, right), the unexpected homogeneous exchange mechanism was revealed. Using the same method, the exchange mechanism in peptide amphiphiles-based nanofibres has been investigated as well.^320^ STORM belongs to the group of SMLM techniques and uses fluorophores that can photoswitch in a suitable buffer. Cy5, for example, can be switched between a fluorescent ‘bright’ and an inactive triplet ‘dark’ state (Fig. 13C, bottom left). The red light that produces fluorescence from Cy5 can also switch it to a long-lived dark state, which can be further stabilised using a buffer. Reaction with oxygen and light exposure can convert Cy5 back to its bright state.^234,327,328^

Later, the same group exploited multicolour interface point accumulation in nanoscale topography (iPAINT) to investigate the structures and dynamics of multicomponent supramolecular fibres *in situ* (Fig. 13D, top right and bottom), which is achievable by simple noncovalent staining without any chemical modification.^319^

Both STORM and PAINT lie within the category of SMLM techniques, but they differ in their mechanisms of switching fluorophores between their ‘ON’ and ‘OFF’ states, which affects the choice of fluorophores. In contrast to STORM, PAINT relies on immobilisation – fast and freely diffusing fluorescent molecules (‘OFF’ state) become visible only when bound to a target (‘ON’ state) (Fig. 13D, top left).^234^

Self-assembly of block copolymers and supramolecular polymerisation leads to the formation of a large variety of complex systems with useful properties and functions. While several synthetic methods have been proposed to generate well-defined self-assembled objects, the construction of assemblies with desired structures in a controlled manner remains a great challenge. Thus, it is of significant importance to develop advanced analytical methods to characterise and gain further understanding of the effects of various parameters, including the concentration and structure of the initial monomer or block copolymer, temperature and solvent type, on the self-assembled structures and self-assembly processes. Fluorescence spectroscopy, such as FRET-based techniques, enables certain insights into self-assemblies, including morphologies, self-assembly kinetics and dynamics. Fluorescence microscopy, especially SRFM, is able to visualise self-assembled structures, to reveal the diversity of structures even within a single object, as well as to unveil the driving force and mechanism of self-assembly. Establishing structure–property relationships at various conditions and revealing the self-assembly mechanism is essential to advance the field, in which fluorescence-based techniques with their high sensitivity, good spatiotemporal resolution and non-invasive characteristic will continue to play a vital role.

## Surfaces, gels and 3D printing

Our ability to synthesise macromolecules with high precision has led to a plethora of soft matter materials with tailored properties.^53,329–333^ The scope of soft matter materials is immense and it would be nearly impossible to discuss every aspect of their application. Therefore, three representative examples – surfaces, gels and 3D printing – are selected herein to discuss the impact of fluorescence-based techniques on their study.

### Surfaces

Surface modification, harnessing the broad chemical and physical properties of macromolecules, is of significant importance for key applications ranging from electronics to biology.^334–337^ With the demand for simple and versatile surface modifications, polymer brushes – more generally defined as thin films of polymer chains tethered at one end to a surface^338^ – have become particularly attractive to achieve finely tuned surface properties including amphiphilic, stimuli-sensitive, antifouling, lubricating and conductive.^339–346^ Two approaches – ‘grafting to’ and ‘grafting from’ – are generally used for the preparation of polymer brushes. The former functioning by grafting pre-formed polymer chains to the surface, while the latter is a bottom-up approach where polymer chains are grown directly from the substrate *via* surface-initiated polymerisation.^99^ Controlled polymerisation methods, including surface-initiated reversible-deactivation radical polymerisation (SI-RDRP),^99^ coupled with various organic reactions provide the ability to control polymer composition, architecture and molecular weight, and thus surface properties and functionalities.^347–353^ Recently, the use of reversible chemistry including supramolecular chemistry and dynamic covalent chemistry, such as Schiff-base chemistry, Diels–Alder chemistry and reversible photochemistry have led to a surge in the development of adaptable and (re)programmable surfaces, with key features including spatial and temporal control over the surface chemistry, reversibility (*e.g.*, writing-erasing-writing) as well as the altered surface properties and functionalities as a result of changes in surface chemistry.^354–360^

Advanced synthetic methods combined with various analytical techniques allow for a better understanding of surface features, such as film thickness, grafting density, chemical composition, molecular weight and dispersity of polymer chains, as well as surface reaction kinetics. These techniques include ellipsometry, AFM, dynamic light scattering (DLS), thermogravimetric analysis (TGA), quartz crystal microbalance, neutron reflectometry, XPS and secondary ion mass spectrometry (SIMS).^99,361–364^

Among these techniques, fluorescence-based techniques enable fast and efficient readout and have been widely exploited to visualise surface grafting of luminescent (co-)monomers, chemical functionalisation with fluorescent dyes as well as the stability of fluorescent films.^345,349,354,360,365,366^ For example, Borozenko *et al.* have utilised total internal reflection fluorescence microscopy (TIRFM) to monitor in real time the detachment of BODIPY-labelled poly(acrylic acid) brushes from glass substrates under varying pH and salt concentrations.^365^ This is a challenging task for conventional surface characterisation techniques such as AFM and ellipsometry, which are limited by the scanning area, measurement speed and adaptability to various environments. TIRFM uses specific optics to achieve total internal reflection of the excitation light at the interface, thus producing an evanescent electromagnetic field. The intensity of the evanescent field decays exponentially with distance from the surface, resulting in the excitation of the region in close proximity to the surface (<200 nm), making TIRFM an powerful analytical technique for thin films.^367^

**Fig. 14:**
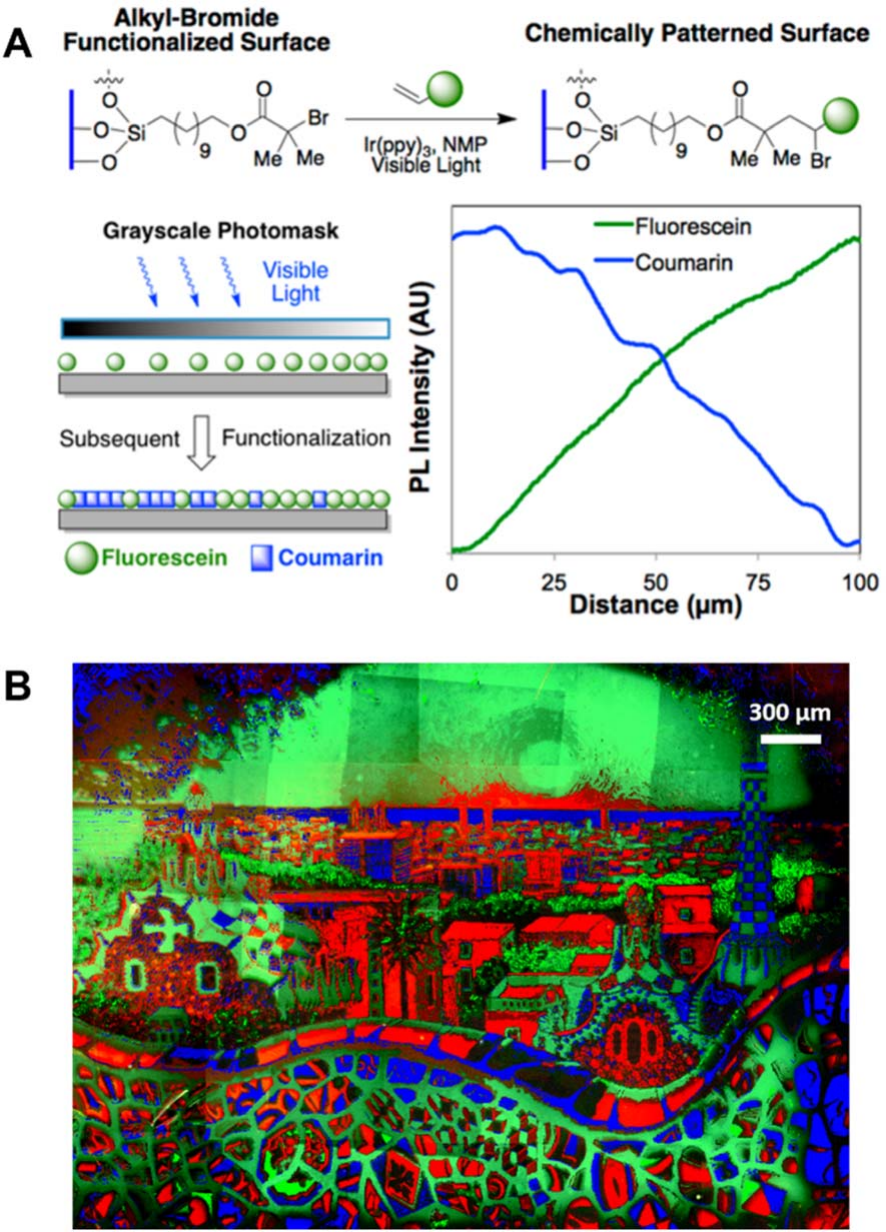
(A) Reaction scheme for the chemical patterning of surfaces, application of a greyscale photomask for chemical concentration gradients and analysis of fluorescence intensity across the chemical concentration gradients surface. Reproduced from ref. 349 with permission from The American Chemical Society, copyright 2013. (B) Three-colour fluorescence microscopy image of a polymer brush surface pattern (Barcelona skyline). Reproduced from ref. 371 (https://doi.org/10.1038/s41467-020-14990-x), under the terms of the CC BY 4.0 license [https://creativecommons.org/licenses/by/4.0/].

In addition to a fast and efficient response, fluorescence microscopy is capable of spatial readout, offering the ability to reveal and distinguish different regions. Surfaces with spatially defined chemical and physical properties are of fundamental importance for studying the structure–property relationships of polymer surfaces and of practical relevance for applications such as microarrays, tissue engineering and microelectronics.^340,341,368^ Hawker and coworkers prepared multifunctional surfaces, nonlinear chemical concentration gradients (Fig. 14A) as well as hierarchical chemical patterns combined photomediated (metal free) SI-ATRP with photomasks.^341,349,352,369^ Braunschweig and colleagues employed surface-initiated thiol–ene photopolymerisation and a photochemical printer based on a digital micromirror device (DMD) to generate high-resolution patterns, circumventing the need for photomasks.^370^ Furthermore, they developed a technique combing a DMD, an atmosphere chamber and microfluidics, capable of generating hypersurfaces, in which more than three properties of each pixel (*x*- and *y*-position, polymer height and monomer composition) can be controlled independently (Fig. 14B).^368,371^ In these studies, confocal microscopy plays a key role for visualisation of different domains of patterns and determining pattern resolutions, while the fluorescence intensity can be used as an indication of the brush height.

**Fig. 15:**
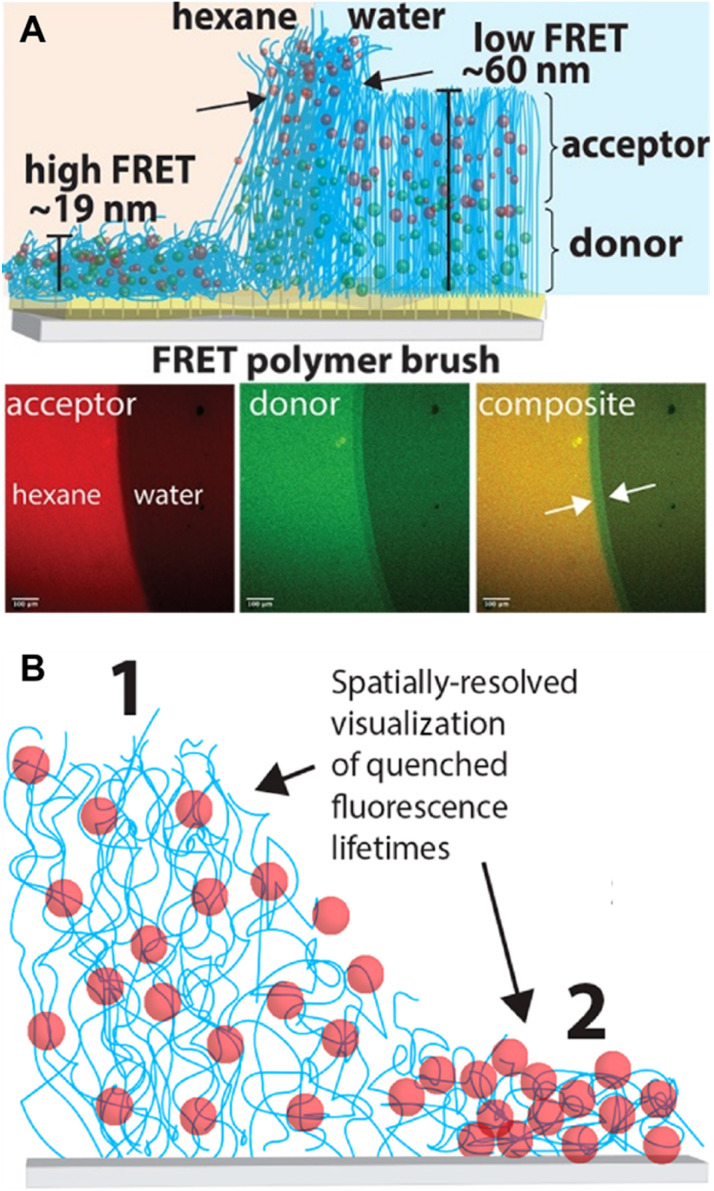
(A) Illustration of polymer brushes with integrated FRET-donor and acceptor and their conformations including heights in hexane and in water (top), and CLSM images of the polymer brush at the interface between hexane and water (bottom). Reproduced from ref. 374 (https://doi.org/10.1002/anie.202104204), under the terms of the CC BY 4.0 license [https://creativecommons.org/licenses/by/4.0/]. (B) Scheme of polymer brushes with covalently tethered fluorophores in swollen and collapsed state. Chain collapse causes the quenching of fluorophores. Reproduced from ref. 388 with permission from The American Chemical Society, copyright 2022.

The spatial read-out capability of fluorescence microscopy further allows to spatially resolve the polymer brush conformation.^372–376^ Polymer brush surfaces with the ability to change the conformation and structure of polymer chains in response to external stimuli hold significant potential for applications in drug delivery, self-healing coatings, sensors and actuators.^339,364^ These external stimuli include temperature, light, applied force and a variety of chemical stimuli, such as solvent type, pH and ionic strength.^356,368,377–383^ Characterisation of surface conformational changes in response to different environmental stimuli is essential for understanding their effects on surface properties. For example, Pemberton and co-workers have investigated the flow penetration depth in poly(*N*-isopropylacrylamide) (PNIPAM) brush films in response to shear flow using TIRFM in combination with FRET by covalently tethering FRET donors to a substrate at the bottom of PNIPAM brush film and adding FRET acceptors in the dynamic flow.^384–386^ Using similar FRET approach, Besford *et al.* studied the molecular transport from aqueous droplets (containing the FRET acceptor) into PNIPAM copolymer brush films incorporating the FRET donor using CLSM instead of TIRFM.^387^ These fluorescence-based measurements demonstrate the changes in polymer brush conformation well. In order to quantify the conformational changes of polymer brushes, Besford *et al.* prepared PNIPAM block copolymer brushes integrating a FRET donor layer followed by a FRET acceptor layer and utilised CLSM to detect the conformational changes of brushes (Fig. 15A, top).^374^ The conformation changes of PNIPAM brushes in response to varying solvents affects the proximity of the donors to the acceptors, thus the polymer brush heights can be correlated to the ratio of fluorescence intensity from donors and acceptors. Alternative method for quantifying polymer brush conformational changes is based on self-quenching of fluorophores covalently tethered to polymer brushes (Fig. 15B).^388^ The collapse of polymer brushes leads to the quenching of these fluorophores, which can be resolved with fluorescence intensity- or lifetime-based techniques (*e.g.*, FLIM), allowing for intensity/lifetime correlation with the heights of brushes. In addition to revealing polymer brush heights, the use of CLSM and FLIM enables the spatial examination of the polymer brush conformation across complex interfaces (Fig. 15A, bottom). In addition, it is worth mentioning that the transduction from conformational changes in stimuli-responsive polymer brushes to the fluorescence signal is particularly attractive for developing surface-based sensors for sensing environmental stimuli, such as temperature, humidity and mechanical force.^374,375,377,388,389^

Examples here focus on planar surfaces, yet fluorescence-based methods are no less important for the characterisation of non-planar surfaces, including nanoparticles, and we refer readers to the relevant literature.^390–393^ High spatiotemporal resolution and non-invasive characteristic of fluorescence-based techniques allow the study of surface grafting kinetics as well as polymer chain dynamics, which will lead to a better understanding of the process and mechanism of surface modification and, in turn, will facilitate the development of more complex polymer surfaces with special properties.

### Gels

Polymer networks, consisting of network junctions and strands linked *via* covalent bonds or non-covalent (supramolecular) interactions, are arguably among the most versatile and widely used soft matter materials.^394^ A large family of polymer networks is the gel, defined as a polymer network that is expanded throughout its entire volume by a gas or more often by a liquid (*e.g.*, hydrogel).^395,396^ Classic industrial applications of polymer gels include hygiene and medical products, such as diapers and contact lenses. Advanced synthetic strategies and methods, including RDRP,^397–400^ dynamic covalent chemistry,^401,402^ mechanically interlocked molecule synthesis and supramolecular chemistry,^403–406^ enable the regulation of the chemistry and structure of polymer networks in gels, critically enriching the variety of gels and endowing them with unique properties, including tuneable mechanical strength, self-healing, self-strengthening and stimuli-responsiveness.^407–415^ These gels hold substantial promise for specialised applications ranging from engineering to biomedical fields, including wearable electronics, soft actuators and robotics as well as tissue engineering.^416–422^ In order to finely control gel materials and exploit the next generation of functional and sustainable gels, a profound understanding of their structures, properties as well as their interaction with the environment is required.

Broadly applicable methods, including rheology, swelling experiments and mechanical tests, provide insights into network structures, but are limited to the macroscopic level, often relying on assumed structure–property relationships. Scattering methods, including neutron scattering and X-ray scattering, have been successfully used to probe the structure of polymer networks, however, limited access and complex experimental set-ups constrain localisation measurements and efforts to obtain information on molecular-scale structural features.^207,394,423^

**Fig. 16:**
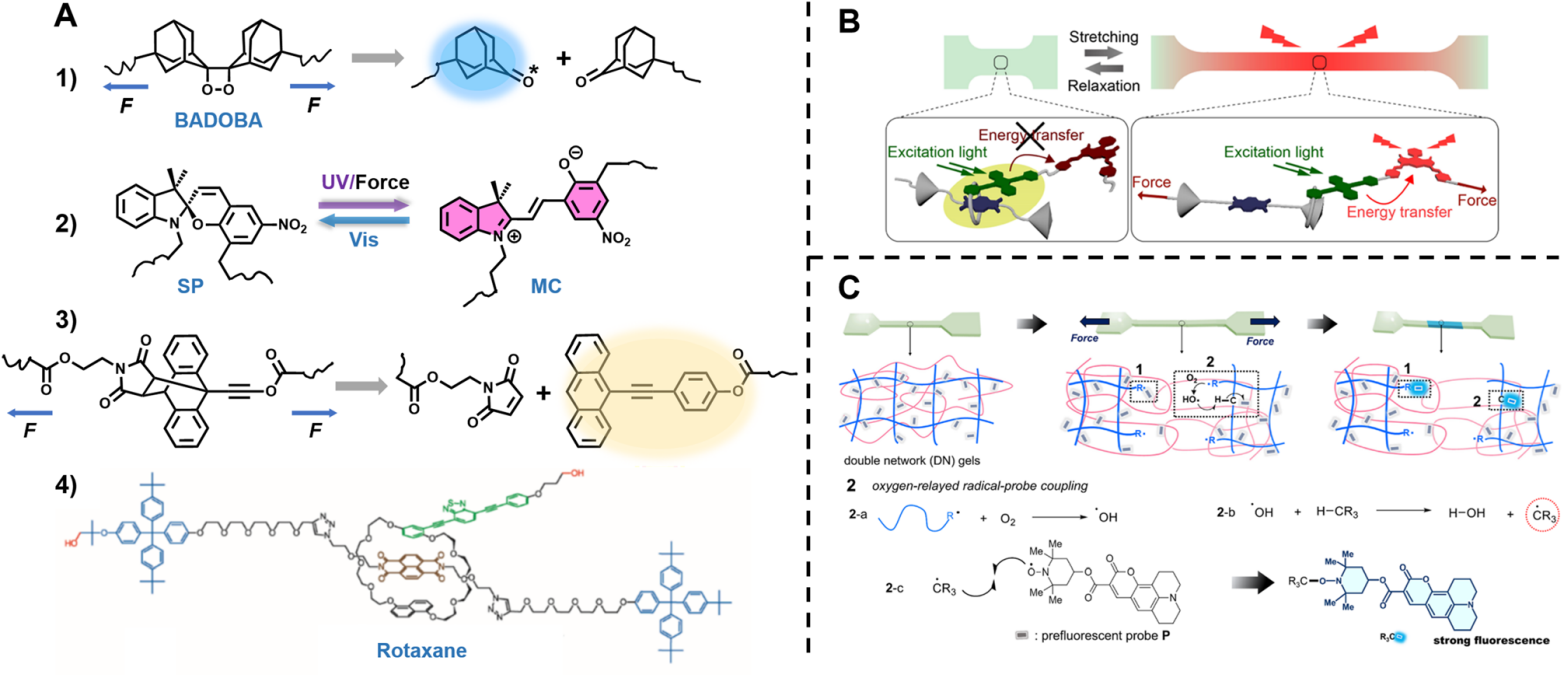
(A) Chemical structures and mechanism of mechanophores. (1) BADOBA. (2) Spiropyran-derivative. (3) A π-extended anthracene adduct. (4) Rotaxane from ref. 441. (B) Illustration of the FRET emission controlled by force on the rotaxane-based supramolecule in stretched hydrogel. Reproduced from ref. 444 (https://doi.org/10.1021/acsami.2c20904), under the terms of the CC BY 4.0 license [https://creativecommons.org/licenses/by/4.0/]. (C) Schematic illustration of the fluorescent reporting of mechanochemical damage in hydrogel, and scheme of oxygen-relayed radical-probe coupling reaction. Reproduced from ref. 445 with permission from The American Chemical Society, copyright 2023.

In recent years, mechanophores – molecules that produce a physical or chemical response to an applied mechanical force – have been employed to study polymer network structures, particularly those that can change colour or emit light, allowing to locate and quantify force in polymer networks at the molecular level.^424–430^ For example, Creton and co-workers have reported the use of mechanophores as cross-linkers, including a dioxetane derivative (bis(adamantyl)-1,2-dioxetane bisacrylate, BADOBA),^431^ a spiropyran derivative (SP)^432,433^ and a Diels–Alder adduct (π-extended anthracene-maleimide adduct),^434,435^ for the spatially resolved visualisation of bond breakage in multi-network elastomers. Under external force, BADOBA cleaves into two adamantanone units, one of which is in the excited state and generates luminescence upon relaxation (Fig. 16(A1)),^431,436,437^ while the colourless SP can be converted to the highly coloured merocyanine (MC) (Fig. 16(A2)),^432,438,439^ and the non-fluorescent π-extended anthracene-maleimide adduct is able to undergo the retro-Diels–Alder reaction, releasing the fluorescent π-extended anthracene moiety (Fig. 16(A3)).^434,435,440^ Instead of using mechanophores, which rely on cleaving covalent bonds, Weder, Sagara and others have employed supramolecular rotaxane-based mechanophores to visualise mechanical forces in elastomers.^441–443^ These mechanophores are composed of a fluorophore-carrying cycle and a dumbbell-shaped molecule containing a matching quencher (Fig. 16(A4)). The displacement of the cycle to the periphery of the dumbbell upon extension leads to fluorescence turn-on. The fluorescence intensity can be further correlated with the applied force.

In a more recent study, Weder and colleagues incorporated a FRET donor and acceptor into the cyclic structure of the rotaxane-based mechanophore, allowing to report the mechanical force applied in polymer hydrogel (Fig. 16B).^444^ In the force free state, the donor emission is suppressed due to the close proximity of donor and quencher. Upon deformation of the hydrogel, the fluorescence emission of the acceptor increases, owing to the separation of the donor from the quencher, thus enabling FRET. Zheng *et al.* established an elegant method to detect and visualise mechanochemical damages in hydrogels by utilising a radical-trapping pro-fluorescent probe (nitroxide radical tethered to coumarin-based luminophore).^445^ The mechanochemical damages often lead to the formation of short-lived free radicals that can either react directly with the probe *via* the radical coupling reaction or undergo an oxygen-relayed radical-transfer reaction first to increase the probability of the coupling reaction, thereby resulting in enhanced fluorescence emission and altered emission colour (Fig. 16C).

Polymer gels, or polymer networks in general, exhibit spatial variations in crosslinking density. These nanoscale structural heterogeneities significantly affect the defining properties of gels (*e.g.*, elasticity, swellability and permeability), and thus tools enabling high spatial resolution characterisation are required to provide more in-depth structural information.^394,425,446^ In contrast to inherently volume-averaging techniques (which lose the characteristics of individual chains within gels), SRFM has demonstrated their potential for new insights, such as the direct visualisation of the positions of crosslinks inside gels.^92,93,208,447–451^

**Fig. 17:**
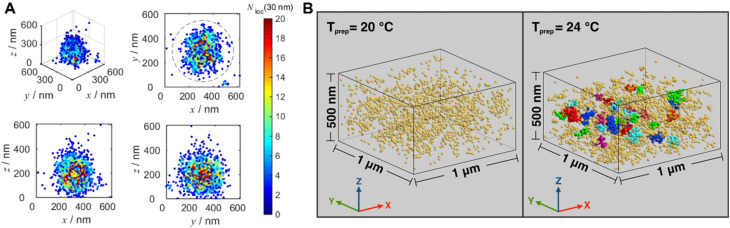
(A) 2D and 3D crosslinker position profiles of an individual microgel, acquired by PALM. Reproduced from ref. 92 with permission from Wiley, copyright 2018. (B) 3D crosslinker distribution profile of a microgel at different temperatures, acquired by W-4PiSMSN. Reproduced from ref. 93 (https://doi.org/10.1039/C8MH00644J), under the terms of the CC BY 3.0 license [https://creativecommons.org/licenses/by/3.0/].

Wöll and his colleagues introduced a diarylethene-based photoswitchable fluorophore as a cross-linker into PNIPAM-based microgels, enabling the visualisation and quantification of cross-linker points using PALM (Fig. 17A).^92^ Ullal and coworkers utilised a SRFM-based technique to map the crosslinking density in PNIPAM microgels as well (Fig. 17B).^93^ They designed a specific cross-linker which can react with rhodamine B or Alexa 647, forming molecules that allow the visualisation by entire cell 4Pi single molecule switching nanoscopy (W-4PiSMSN), a type of optical nanoscopy that enables imaging 3D structures at 10–20 nm resolution. Both studies show the cross-linker density within a microgel decreases as a function of the distance from its centre, revealing the heterogeneity of the polymer networks constituting microgels.

Due to the complexity of their structures, gels often exhibit sophisticated behaviours and properties. Characterisation of gels with molecular precision is challenging, but critical for the in-depth understanding of structure–property relationships and further development of gels with specific functions. Judicious selection of mechanophores enables the observation of forces in polymer networks and powerful SRFM techniques allow the visualisation of crosslinking networks. Both methods can be exploited to shed new light on polymer gels and will undoubtedly be extended to a wide range of gels and provide even more quantitative information in the future.

### 3D printing

Since the pioneering work by Hull in the early 1980s,^452^ 3D printing has attracted enormous attention and has been extensively applied in a wide range of fields from architecture, automotive to medicine.^453–455^ The ability to translate computer-aided design into complex 3D physical objects has revolutionised manufacturing and has had an immeasurable impact on modern society. Among various 3D printing techniques (fused filament fabrication, inject printing, *etc.*), light-based techniques, including direct laser writing (DLW) or multi-photon stereolithography enable the fabrication of well-defined, high-resolution 3D objects by spatiotemporal control of reaction.^456–459^ Light-based 3D printing generally involves converting reactive liquid resins into solid materials by photopolymerisation, such as free radical, cationic and thiol–ene polymerisation, providing 3D objects with diverse chemical and physical properties.^102,460–464^

Recently, a variety of (photo)chemical reactions opened new opportunities for 3D printing. Photo-RDRP confers the ability to control the nanostructures and mechanical properties of 3D printed objects.^465^ The living characteristics of polymer chains formed by RDRP allow the post-modification and self-healing of objects.^52,56,57^ Photoresists that possess labile linkage (*e.g.*, coumarin derivatives) or that can undergo (photo)chemical ligation (*e.g.*, photoenol moieties reacting with maleimide) can impart responsive behaviour, such as degradability and programmability, onto the printed structures.^51,55,58,466–468^

The use of multiple colours of light instead of a single colour can further exploit the vast potential of light-based 3D printing. Two-colour printing is generally based on synergistic, orthogonal or antagonistic photochemistry.^469^ Synergistic photochemistry requires the simultaneous irradiation with two distinct colours to trigger a reaction, in an orthogonal system two reactions are induced by two wavelengths and proceed independently, while antagonistic implies opposing reaction processes triggered separately by two colours of light. Two-colour systems not only allow access to new and complex materials with unique properties, but also facilitate the development of superior printing approaches and techniques, such as light-sheet printing,^470^ xolography,^471^ multimaterial printing^53,472^ and STED-based nanoprinting.^473,474^ In addition to multi-colour printing, research on 4D printing is growing substantially. Incorporating an additional dimension – time – into 3D printing, 4D printing describes the capability of objects to transform over time directly off the print bed,^475^ often requiring stimuli-responsive materials, such as hydrogels,^476,477^ liquid crystal elastomers^478^ and shape memory polymers.^479–481^

With the flourishing development of 3D printing towards objects with highly complex materials and structures and with high-resolution features, it is of vital importance to characterise them at the micro- and molecular-scale and further understand how the macroscopic properties are influenced by the molecular-scale polymer structures and possible heterogeneities, thus benefiting control of chemical reactions and resulting materials properties in 3D printing with high precision. Several advanced techniques have been applied to characterise the local properties of 3D-printed objects, including AFM quantitative nanomechanics (QNM) and NanoDMA to determine local mechanical properties,^482,483^ Micro-FTIR and time-of-flight SIMS (ToF-SIMS) to provide chemical information at microscale^56,484^ as well as X-ray micro-computed tomography (XRM) to visualise 3D internal structures.^485^ However, these techniques are either restricted to surface measurements or have limited spatial resolution. Thus, it is challenging but critical to develop alternative methods to investigate internal polymer structures at the microscopic and molecular scale. Fluorescence-based techniques have demonstrated strong abilities in various aspects of polymers and soft matters and are therefore a high-potential candidate for studying 3D printed soft matter structures.

**Fig. 18:**
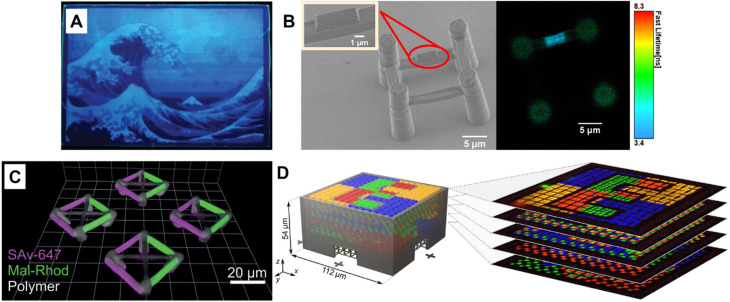
(A) Fluorescence image of a surface pattern (The Great Wave off Kanagawa) on a 3D printed rectangular prism made by type I photoinitiated RAFT polymerisation. Reproduced from ref. 486 with permission from Wiley, copyright 2021. (B) SEM and FLIM images of a surface-modified 3D ‘bridge’ microstructure 3D microstructure made by photoiniferter-RAFT polymerisation and surface functionalisation made from monomers without photoinitiators. Reproduced from ref. 56 with permission from Wiley, copyright 2021. (C) 3D CLSM images showing spatially resolved dual surface functionalisation using photo-ligation reactions on a 3D printed scaffold (green parts, based on photoenol ligation). Reproduced from ref. 51 with permission from Wiley, copyright 2016. (D) Design (left) and CLSM image stack (right) of a multimaterial microstructure made from one non-fluorescent and four fluorescent photoresists. Reproduced from ref. 489 (https://doi.org/10.1126/sciadv.aau9160), under the terms of a CC BY 4.0 license [https://creativecommons.org/licenses/by/4.0/].

Fluorescence techniques, particular CLSM, have been used to demonstrate the living properties of 3D structures generated *via* photo-RDRP^54,56,486,487^ (Fig. 18A and B), to demonstrate the capability of photoenol-based photochemical ligation reactions for spatially resolved surface functionalisation of 3D microstructures^51,488^ (Fig. 18C) as well as to highlight the ability of a microfluidic-based 3D laser microprinting to integrate multiple materials^489^ (Fig. 18D). The successful proof of these three listed concepts in the field of 3D printing is attributed to the spatially resolved capabilities of fluorescence techniques.

**Fig. 19:**
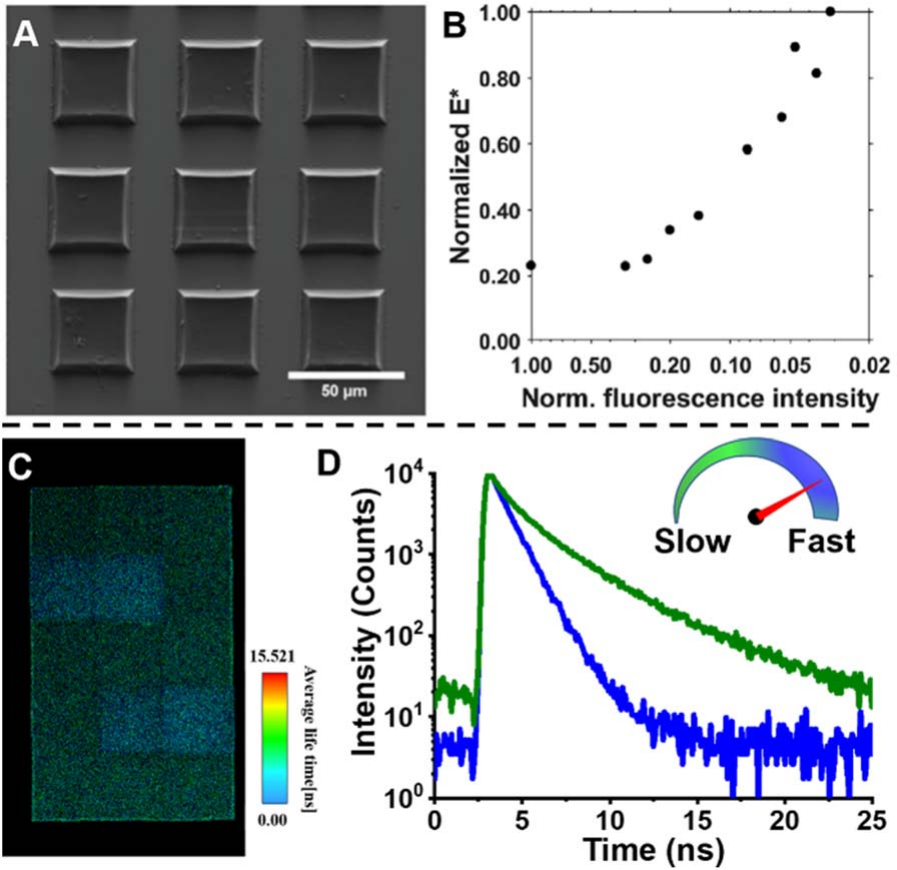
(A) SEM image of microstructures containing only anthracene dimers as monomers. Reproduced from ref. 482 with permission from Wiley, copyright 2019. (B) Normalised complex modulus (E*) determined by NanoDMA *versus* normalised fluorescence intensity of the structures shown in (A) determined by CLSM. Reproduced from ref. 482 with permission from Wiley, copyright 2019. (C) FLIM image of a microstructure made from two different photoresists, containing BODIPY-C12. Reproduced from ref. 490 with permission from The Royal Society of Chemistry, copyright 2022. (D) Fluorescence decay curves of BODIPY-C12 in the same microstructure as (C) showing BODIPY-C12 in different viscosity environments. Reproduced from ref. 490 with permission from The Royal Society of Chemistry, copyright 2022.

Furthermore, fluorescence-based techniques allow for the study of polymer structures in 3D objects at the micro- and molecular scale, thus further establishing the relationship between polymer structures and their properties. Our group has introduced an anthracene dimer-containing photoresist for DLW and succeeded to produce microstructures with adaptive mechanical properties by taking advantage of the [4 + 4] photodimerisation of anthracene (Fig. 19A).^482,484^ Under visible light irradiation, concomitant with the change in mechanical properties, the fluorescence intensity of microstructures decreases, indicating an increasing amount of photodimerisation, as the anthracene dimer does not display fluorescence. The direct correlation between fluorescence and mechanical properties (Fig. 19B) allows the fluorescence-read out for mechanical properties of microstructures. As noted, the fluorescence intensity decreases before the change in mechanical properties, as fluorescence reflects changes on the molecular level, indicating the high sensitivity of fluorescence-based techniques for the investigation of polymer structures in 3D-printed objects. Very recently, Wu, Belqat *et al.* have utilised FLIM to investigate the microenvironment in two-photon fabricated microstructures employing the fluorescent molecular rotor – BODIPY-C12 – as a viscosity probe (Fig. 19C).^490^ The bi-exponential fluorescence decays obtained from fluorescence lifetime measurements in the microstructures show that BODIPY-C12 is present in different local micro-viscosities, indicating the heterogeneity in the fabricated microstructures. The dependence of fluorescence lifetime on BODIPY-C12 on the local viscosity has been further exploited to distinguish different materials in multi-material printed objects (Fig. 19C and D) – a strategy that opens new perspectives for probing 4D and multi-material characteristics of 3D objects at the micro- and molecular level.

The stimuli-responsiveness of objects is the key feature of 4D printing. Conceivably, the integration of fluorescent probes, including viscosity, humidity probes and mechanoluminophores, into 3D-printed objects, allows to reveal the local microenvironment within the objects, which defines their macroscopic properties and their transformation in response to stimuli. Advanced fluorescence techniques, such as FLIM, STED and SMLM combined with suitable fluorescent probes can provide a wealth of information on polymer structures, including heterogeneity, crosslinking points as well as polymer chain orientation. In the realm of 3D printing, the study of structure–property relationships at the molecular level is still in its infancy, yet is critical to achieve 3D objects with desired and tailored properties, and we recommend that fluorescence-based techniques play a significant future role. Furthermore, the capability of *in situ* and real-time response afforded by fluorescence-based techniques will certainly be exploited in the future to understand polymerisation kinetics during 3D printing.

## Summary and outlook

The field of polymer science has made substantial progress over the past few decades. The fusion of modern polymerisation methods with advanced organic chemistry has allowed us to access diverse polymers with well-defined architectures and functionalities, including sequence-defined polymers. The incorporation of supramolecular chemistry has further expanded the scope of macromolecules regarding their morphology and topology. These advances have led to significant developments of soft matter materials, including surface-grafted polymer brushes, responsive gels, delivery vectors and sensors as well as a vibrant field – 3D printing. To gain an accurate understanding of these complex materials, the development of advanced analytical characterisation methods is undoubtedly critical. Fluorescence-readout has emerged as a powerful characterisation tool, providing new and deep insights into macromolecules and soft matter materials, enabling to visualise and monitor various polymerisation processes *in situ*, to quantify the ratio of different arms in miktoarm star polymers and the crosslinking points in polymer networks, to visualise a single polymer chain in its native environment, to study kinetics and dynamics of polymer self-assembly as well as to spatially resolve the conformation of polymer brushes on surfaces, to observe forces in gels with molecular precision and to reveal the heterogeneity in printed microstructures, attributed to the successful integration of (pro)fluorophores and implementation of advanced fluorescence techniques. Building on these successes, the field holds specific key future opportunities, including but not limited to: (i) *in situ* monitoring of polymerisation processes starting from the initiation stage, and providing detailed spatial information; (ii) exploiting fluorescence-based techniques for more complex architectures and assemblies to determine structure–property relationships; (iii) providing quantitative information on polymer conformation in their native environments for the validation and generation of accurate models; (iv) applying advanced fluorescence techniques to a wider range of polymer systems in surfaces and gels, not limited to model polymers; (v) associating fluorescent probes with advanced fluorescence techniques to reveal micro- and molecular structures and their environment in gels and 3D-printed objects; (vi) studying polymerisation kinetics of surface grafting and 3D printing in an *in situ*, real time manner and (vii) exploiting (orthogonal) fluorescence-readouts and tracing providing molecular identity information in an efficient and optically readable fashion. Fluorescence-readout fuels the dream of all polymer chemists – control of soft matter material properties with precision on the molecular level. However, reaching our aspirations will undoubtedly necessitate coordinated endeavours across disciplines including chemistry, physics, engineering and material science.

## Author contributions

The current review was written with contributions from both authors.

## Conflicts of interest

There are no conflicts to declare.

## Supplementary Material
